# Comprehensive bioinformatics analysis of acquired progesterone resistance in endometrial cancer cell line

**DOI:** 10.1186/s12967-019-1814-6

**Published:** 2019-02-27

**Authors:** Wenzhi Li, Shufen Wang, Chunping Qiu, Zhiming Liu, Qing Zhou, Deshui Kong, Xiaohong Ma, Jie Jiang

**Affiliations:** 1 Department of Obstetrics and Gynecology, Qilu Hospital of Shandong University, No. 107 Wenhua Road, Jinan, 250012 Shandong China; 2 Department of Obstetrics and Gynecology, Ningjin County Planned Parenthood Maternal and Child Health Care Service Center, Dezhou, 253400 Shandong China; 3 Department of Obstetrics and Gynecology, First Affiliated Hospital of Wenzhou Medical University, Wenzhou, 325000 Zhejiang China

**Keywords:** Progesterone resistance, Endometrial carcinoma, Bioinformatics, Progesterone receptor

## Abstract

**Background:**

Progesterone resistance is a problem in endometrial carcinoma, and its underlying molecular mechanisms remain poorly understood. The aim of this study was to elucidate the molecular mechanisms of progesterone resistance and to identify the key genes and pathways mediating progesterone resistance in endometrial cancer using bioinformatics analysis.

**Methods:**

We developed a stable MPA (medroxyprogesterone acetate)-resistant endometrial cancer cell subline named IshikawaPR. Microarray analysis was used to identify differentially expressed genes (DEGs) from triplicate samples of Ishikawa and IshikawaPR cells. PANTHER, DAVID and Metascape were used to perform gene ontology (GO), Kyoto Encyclopedia of Genes and Genomes (KEGG) pathway enrichment analysis, and cBioPortal for progesterone receptor (PGR) coexpression analysis. GEO microarray (GSE17025) was utilized for validation. The protein–protein interaction network (PPI) and modular analyses were performed using Metascape and Cytoscape. Further validation were performed by real-time polymerase chain reaction (RT-PCR).

**Results:**

In total, 821 DEGs were found and further analyzed by GO, KEGG pathway enrichment and PPI analyses. We found that lipid metabolism, immune system and inflammation, extracellular environment-related processes and pathways accounted for a significant portion of the enriched terms. PGR coexpression analysis revealed 7 PGR coexpressed genes (ANO1, SOX17, CGNL1, DACH1, RUNDC3B, SH3YL1 and CRISPLD1) that were also dramatically changed in IshikawaPR cells. Kaplan–Meier survival statistics revealed clinical significance for 4 out of 7 target genes. Furthermore, 8 hub genes and 4 molecular complex detections (MCODEs) were identified.

**Conclusions:**

Using microarray and bioinformatics analyses, we identified DEGs and determined a comprehensive gene network of progesterone resistance. We offered several possible mechanisms of progesterone resistance and identified therapeutic and prognostic targets of progesterone resistance in endometrial cancer.

**Electronic supplementary material:**

The online version of this article (10.1186/s12967-019-1814-6) contains supplementary material, which is available to authorized users.

## Background

Endometrial cancer (EC) is the most common gynecologic malignancy in developed countries. According to cancer statistics in China, there were 634 per 100,000 newly diagnosed and 21.8 per 100,000 mortalities in 2017. EC had a significant upward trend in age-standardized incidence rates [1].

Approximately 80% of EC cases are type I endometrial cancer, which are highly associated with prolonged unopposed estrogen action and insufficient progesterone. Common progestin therapy drugs including medroxyprogesterone acetate (MPA) and megestrol acetate (MA), have been used as a conservative treatment for stage 1A low-grade EC patients who desire to preserve fertility or patients with comorbidities who are unsuitable candidates for operation. Recurrent endometrial cancer patients can also receive hormonal therapy. Some studies have indicated that synthetic progesterone is effective for stage 1A EC [2–7]. However, approximately 30% of early stage 1A EC patients never responded to treatment or only exhibit a temporary response when treated as primary therapy [2, 7]. For advanced cases, the response rate is only 20–40% [8]. Although 70% of early patients respond to treatment, 57% of these patients experience recurrence. When treated by MPA again after diagnosis of recurrence, 63% were nonresponsive [7]. All of the above findings indicate that de novo or acquired progestin resistance is a major clinical problem.

Several studies have investigated the mechanism of progesterone resistance in EC, but the precise mechanism remains unknown. When progesterone resistance occurs, progestin can promote proliferation and invasiveness rather than inhibit growth and promote apoptosis of cancer cells [9]. Previous mechanistic explanations include imbalance of ER and PR subtypes [10], overexpression of EGFR and activation of TGF-EGFR signaling [11–13], activation of the PI3K/AKT/mTOR pathway [14, 15], dysregulation of Nrf2-survivin and overexpression of survivin [16]. We previously developed a stable MPA-resistant Ishikawa cell and confirmed that SIRT1/FoxO1/SREBP-1 acts as a pathway targeting PR that is involved in the development of progesterone resistance in endometrial cancer cell [17]. However, current research does not sufficient explain the key genes that cause the down regulation of PR in progesterone resistance and molecular network involved in progesterone resistance.

Microarray analysis is a high-throughput approach that has been used more than 10 years. Bioinformatics analysis of microarray data, including gene clustering, gene ontology and pathway analysis, has shown great significance to identify potential key genes and pathways within a complex disease or biological process [18]. No microarray profiling study of progesterone resistance within endometrial cancer has been performed. In this work, we performed microarray analyses to comprehensively analyze the expression profile of progesterone resistance. In total, 821 DEGs, 7 PGR coexpressed genes, 8 hub genes and 4 MCODEs were found and further analyzed by GO, pathway enrichment and PPI analyses. We found that lipid metabolism, the immune system and inflammatory response and the extracellular environment may be crucial in progesterone resistance. These hub genes may help us identify novel biomarkers and treatment targets for progesterone resistance in the future.

## Methods

### Cell culture

Ishikawa EC cells were purchased from American Type Culture Collection (ATCC; Rockvile, MD, US). MPA-resistant Ishikawa cell, which is referred to as IshikawaPR was established as previously described [17]. Ishikawa and IshikawaPR were routinely grown in RPMI 1640 (HyClone, USA) containing 10% fetal bovine serum (FBS) at 37 °C in a 5% CO_2_ humidified atmosphere. IshikawaPR cell was routinely cultured in 10 μM MPA to maintain resistance.

### Microarrays and bioinformatics analysis

Microarray analysis was performed using triplicate samples of parental Ishikawa and IshikawaPR cells. Total RNAs were prepared and subjected to Agilent Human 4 × 44 K Gene Expression Microarrays according to the manufacturer’s instructions (Agilent) after quality control assessment. Sample labeling and microarray hybridization were performed using the Agilent One-Color Microarray-Based Gene Expression Analysis protocol (Agilent Technology). Array images were acquired and analyzed using the Agilent Feature Extraction software (version 11.0.1.1). Normalization and subsequent analytical data processing were performed using the GeneSpring GX v12.1 software package. Genes for which at least 3 out of 6 samples exhibited detection were chosen for further data analysis. The raw data.tar has been submitted to GEO (Series GSE121367 https://www.ncbi.nlm.nih.gov/geo/query/acc.cgi?&acc=GSE121367) and will be released until Dec 31, 2019.

Differential expressed genes (DEG) with statistical significance were identified through volcano plot filtering. The thresholds for DEG were |logFC| ≥ 4.0 and *p* value < 0.05 (Additional file 1: Table S1). Hierarchical clustering was performed using Morpheus (https://software.broadinstitute.org/morpheus/). GO analysis and pathway enrichment were performed using multiple databases, including Gene Ontology consortium (http://www.geneontology.org/), PANTHER (http://www.pantherdb.org/), DAVID (https://david.ncifcrf.gov/), Metascape (http://metascape.org/gp/index.html#/main/step1), AmiGo 2 (http://amigo.geneontology.org/amigo/landing), Funrich and KEGG (http://www.genome.jp/kegg/), using p < 0.05 as the cut-off criterion.

### PGR coexpression analysis and validation by bioinformatics

Assessment of the coexpression genes of PGR was performed using the cBioPortal database (http://www.cbioportal.org). The data obtained were RNA-Seq data from TCGA database that included 549 endometrial cancer tissues. Pearson’s correlation score and Spearman score (≥ 0.3 was considered positively correlated and ≤ − 0.3 was considered negatively correlated with PGR) were used to select PGR coexpressed genes. To predict the target genes that were changed in IshikawaPR, we use FunRich to identify the overlapping genes between DEGs and PGR coexpressed genes.

For validation of target genes, the gene expression profile result, GSE17025, deposited by Day et al. [19] was used. The gene expression profile has 91 Stage I endometrial cancer patients and 12 postmenopausal healthy tissues. Then we calculated Pearson and Spearman score between target genes and PGR and compared the expression of target genes among healthy and different type of endometrial cancer tissues (p < 0.05 as cut-off criterion) using GraphPad Prism 7.0 and SPSS 22.0 software. Kaplan–Meier curves for target genes were generated with the online tool Kaplan–Meier Plotter (http://www.kmplot.com/). A total of 542 RNA-seq data samples of uterine corpus endometrial carcinoma were interrogated. The patients were split into 2 groups (high vs. low) based on the expression level.

### Validation of target genes by real-time PCR

Total RNA was extracted from Ishikawa and IshikawaPR cells using TRIzol reagent (Invitrogen, Carlsbad, CA) according to the manufacture’ instructions. Total RNA (3 μg) was reverse transcribed using the M-MLV reverse transcriptase (Cat no. C28025-011, Invitrogen, China). Then RNA expression level of detected genes were quantified using an ABI Prism 7500 Sequence Detection System (Applied Biosystems, USA) with SYBR Green Master Mix (Takara, Japan) in a 20 µl reaction mixture. The primers were synthesized by Sangon Biotech Corporation and presented in Additional file 2: Table S2. Experiments were repeated in triplicate.

### Protein–protein interaction (PPI) network construction analysis

STRING and Cytoscape were used to establish a PPI network, and proteins with degree > 1 were selected. The network analyzers CentiScape and MCODE of Cytoscape software were used to analyze the topology property of the network. Genes with a degree of connectivity > 15 were defined as hub genes. MCODEs were extracted when the Node score cut-off was 0.2 and K-core was 2.

## Results

### Identification of DEGs from parental and IshikawaPR cells

**Fig. 1:**
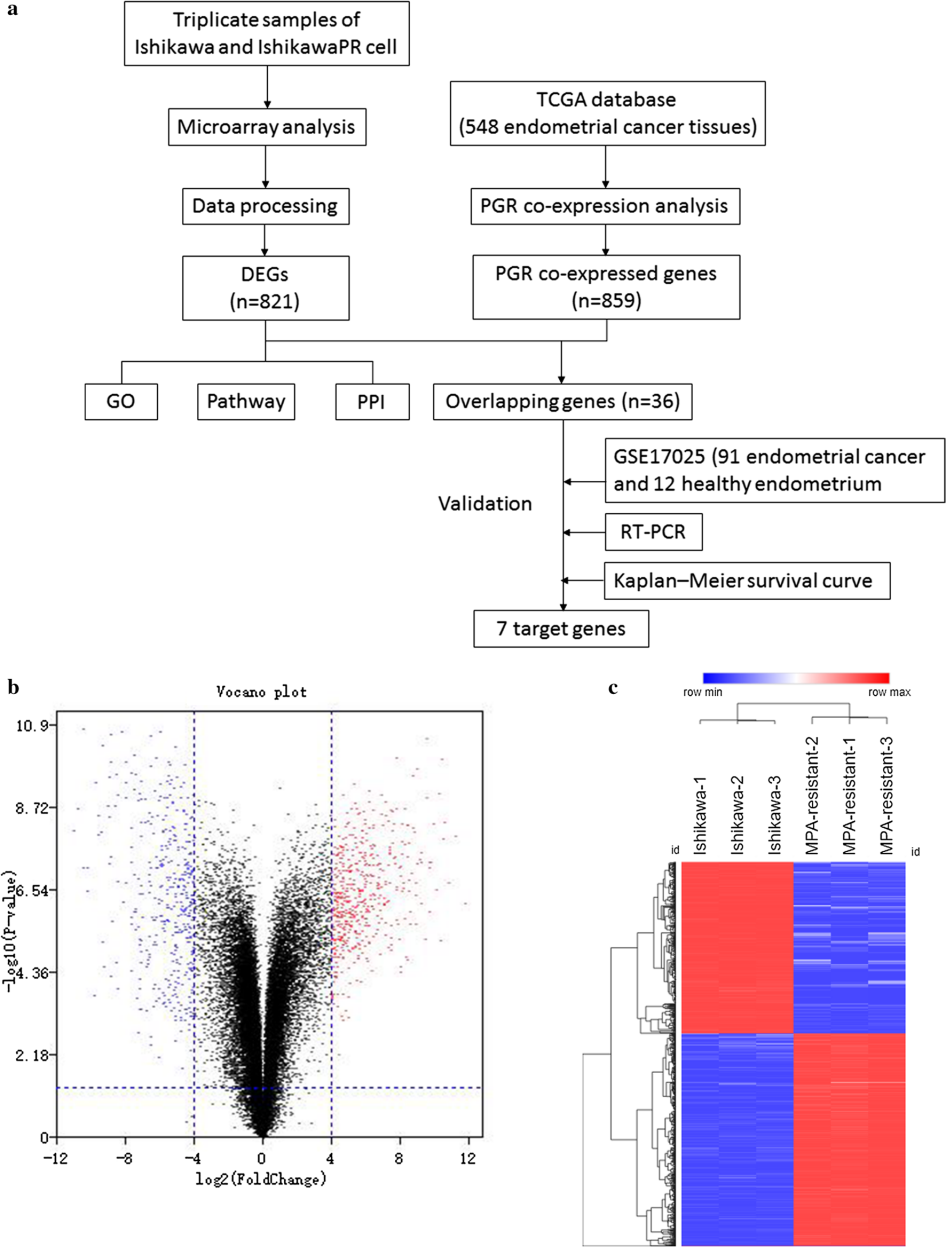
Identification of differentially expressed genes (DEGs) in MPA-resistant Ishikawa cell using Morpheus Website. **a** Overall design of the study. **b** Volcano plot of the 27831 expressed genes. Red color represented up-regulated genes in IshikawaPR cell than Ishikawa cell and blue color represented down-regulated genes. **c** Clustering heatmap of the 821 genes exhibiting significantly differential expression, statistically significant DEGs were defined as |Log2Foldchange| > 4 and p-value < 0.05. GO: gene ontology; PPI, protein–protein interaction; TCGA, The Cancer Genome Atlas; PGR, progesterone receptor; RT-PCR, real-time polymerase chain reaction

**Fig. 2:**
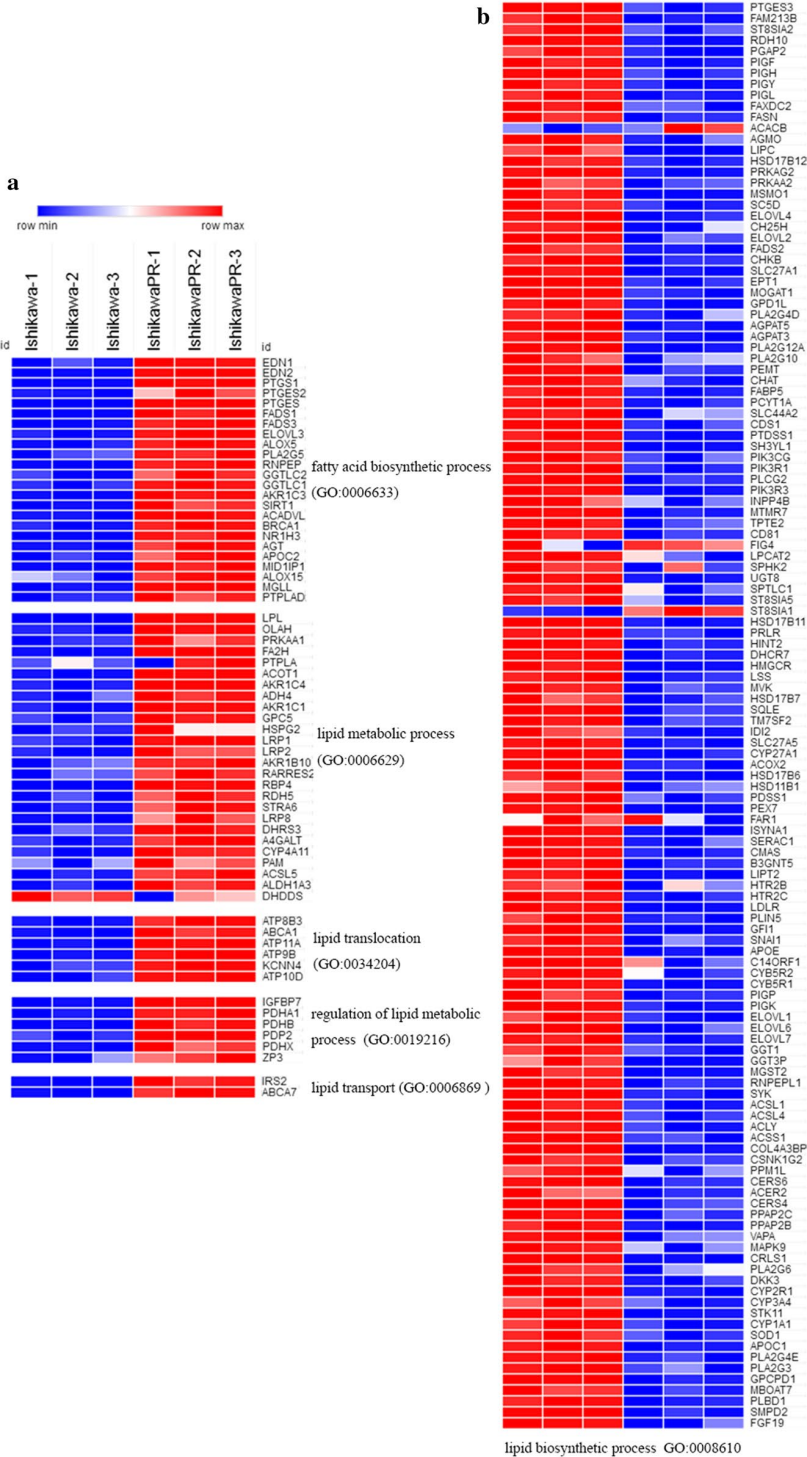
Heatmap of genes that are related to lipid metabolic and biosynthetic process in IshikawaPR and Ishikawa cell. Genes shown in red are up-regulated and blue are down-regulated in IshikawaPR cell. **a** Heat map of genes related to “lipid metabolic process” (fatty acid biosynthetic process (GO: 0006633), lipid metabolic process (GO: 0006629), lipid translocation (GO: 0034204), regulation of lipid metabolic (GO: 0019216), lipid transport (GO: 0006869). **b** Heat map of genes related to “lipid biosynthetic process (GO: 0008610)”

We performed microarray analysis to identify differentially expressed genes between parental and IshikawaPR cells (Fig. 1). Volcano plots displayed the distribution of the 27,831 expressed genes (Fig. 1b). Using |LogFC| > 4 and p-value < 0.05 as cut-off criteria, 821 DEGs were extracted, including 453 upregulated and 368 downregulated genes (Fig. 1c, Additional file 1: Tables S3, Additional file 3: Table S2). In total, 760 genes were coding RNA, and 61 genes were noncoding RNA, including 30 long noncoding RNA (LncRNA), 19 noncoding RNA (NR) and 12 uncharacterized RNA. Using the Morpheus website, we developed a clustering heatmap of the DEGs (Fig. 1c). Interestingly, based on the heatmap of genes involved in lipid metabolism and biosynthetic processes, we found that lipid metabolism process-related genes, including fatty-acid biosynthetic processes, lipid translocation, regulation of lipid metabolic processes and lipid transport, were mostly upregulated, lipid biosynthetic process-related genes were downregulated in IshikawaPR cell (Fig. 2, Additional file 4: Table S4). RNA expression level changes in lipid metabolism-related genes in IshikawaPR cells indicated that reduced lipid biosynthesis and increased metabolism may participate in the mechanism of progesterone resistance.

### Gene ontology analysis of DEGs

**Fig. 3:**
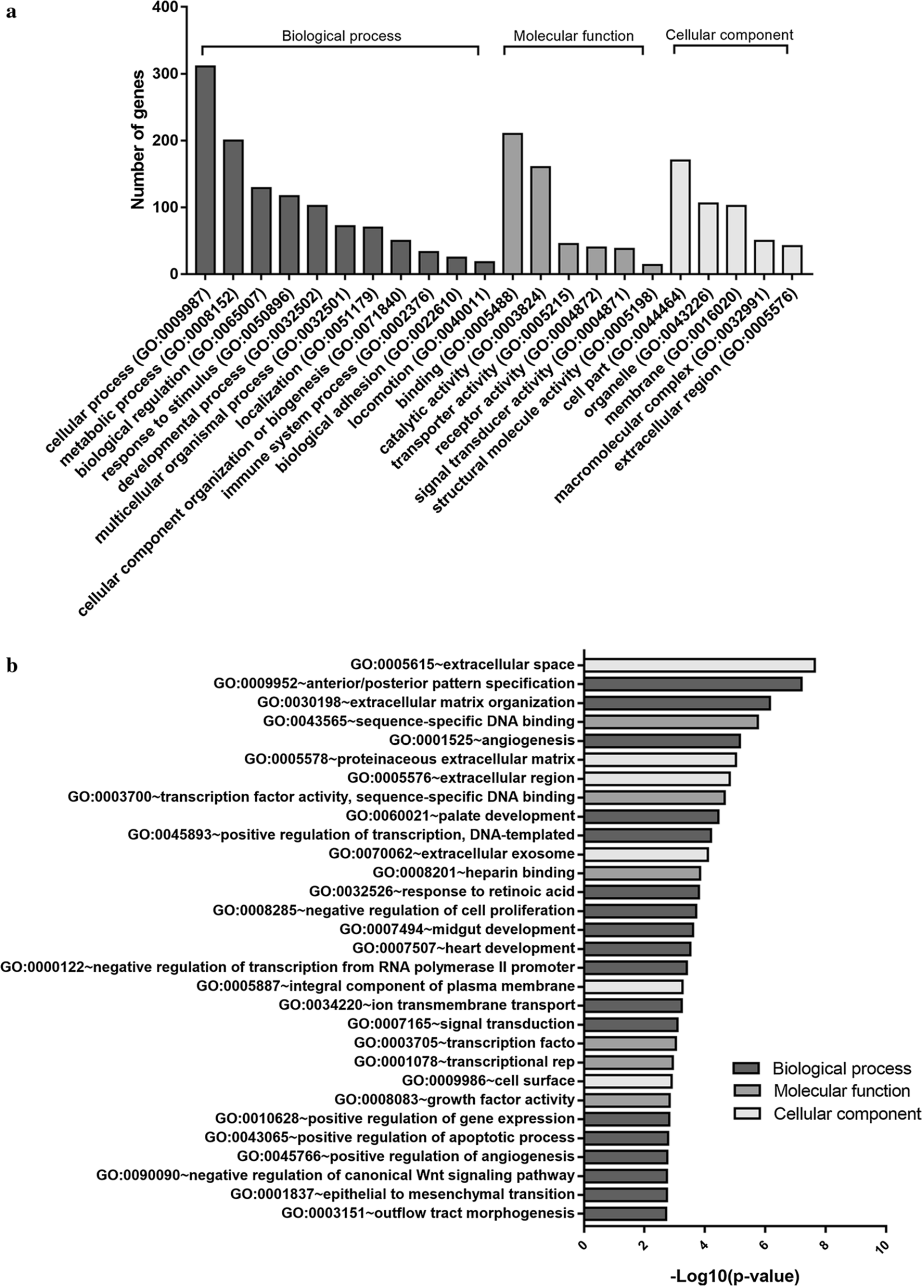
Gene ontology (GO) enrichment of 821 DEGs in IshikawaPR cells. **a** Sorted by descending order of the number of genes associated with the listed GO ID. **b** Sorted by descending order of −Log10(p-value) for the GO enrichment terms

To gain more biological insight, we performed gene oncology (GO) enrichment analysis using multiple online databases, including PANTHER, DAVID, and Metascape. The DEGs were classified into three functional groups: biological process (BP), molecular function (MF) and cellular component (CC). The most enriched BP functions were cellular processes (311 genes) and metabolic processes (200 genes). For MF, binding (210 genes) and catalytic activity (160 genes) were the most enriched. In the clusters of CC, cell part (170 genes) and organelle (106 genes) genes were the most enriched (Fig. 3a). As shown in Table 1 and Fig. 3b, in the biological process group, upregulated genes were mainly enriched in anterior/posterior pattern specification, response to retinoic acid, extracellular matrix organization, antigen processing and presentation of peptide antigens via MHC class I, down-regulated genes were mainly enriched in palate development, bicellular tight junction assembly, extracellular matrix organization, and angiogenesis. In the molecular function group, upregulated genes were mainly enriched in sequence-specific DNA binding. In the cellular component group, upregulated genes mainly enriched in extracellular space, extracellular region and the cell surface, downregulated genes were mainly enriched in lateral plasma membrane, ruffle membrane and bicellular tight junction. These results showed that DEGs were mainly enriched in extracellular environment, cell junction, and immune system.

**Table 1:** Top 10 GO enrichment analyses of up-regulated and down-regulated DEGs

Category	Term	Count	p-value
Up-regulated			
BP	GO:0009952 ~ anterior/posterior pattern specification	14	1.65E−08
CC	GO:0005615 ~ extracellular space	56	3.21E−07
MF	GO:0043565 ~ sequence-specific DNA binding	30	2.32E−06
BP	GO:0032526 ~ response to retinoic acid	8	2.57E−05
CC	GO:0005576 ~ extracellular region	57	3.21E−05
CC	GO:0005578 ~ proteinaceous extracellular matrix	17	1.07E−04
BP	GO:0048704 ~ embryonic skeletal system morphogenesis	7	1.80E−04
CC	GO:0009986 ~ cell surface	25	2.58E−04
BP	GO:0030198 ~ extracellular matrix organization	14	3.68E−04
BP	GO:0002474 ~ antigen processing and presentation of peptide antigen via MHC class I	6	4.30E−04
Down-regulated			
BP	GO:0042472 ~ inner ear morphogenesis	6	0.001957
BP	GO:0060021 ~ palate development	7	0.001967
BP	GO:0070830 ~ bicellular tight junction assembly	5	0.002114
BP	GO:0030198 ~ extracellular matrix organization	11	0.002153
CC	GO:0016328 ~ lateral plasma membrane	6	0.002206
CC	GO:0032587 ~ ruffle membrane	7	0.003009
CC	GO:0005923 ~ bicellular tight junction	8	0.003557
BP	GO:0001525 ~ angiogenesis	11	0.005387
BP	GO:0007165 ~ signal transduction	33	0.005738
BP	GO:0007156 ~ homophilic cell adhesion via plasma membrane adhesion molecules	9	0.0061

Count: number of DEGs that hit in the term

### Encoded protein class and KEGG pathway enrichment analysis of DEGs

**Fig. 4:**
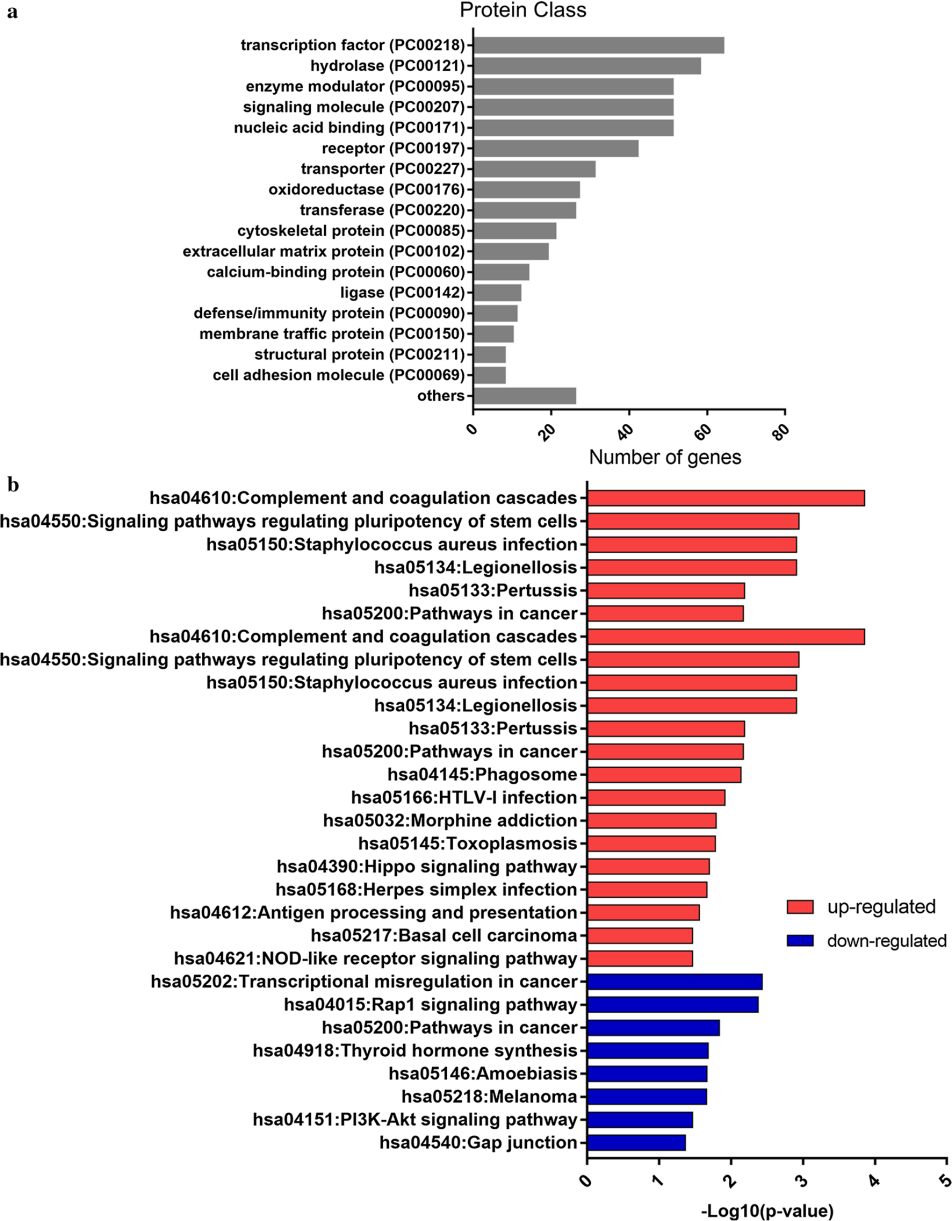
Protein classes of DEGs and KEGG pathway enriched by up- and down- regulated genes respectively. **a** The proteins of DEGs were classified according to its function. Protein classes was sorted by number of genes contained. **b** Pathway enriched by up- and down-regulated genes respectively and sorted by value of –Log10(p-value)

To further comprehend the proteins function of those DEGs, we analyzed the protein classes of DEGs (Fig. 4a). DEGs encoded proteins were mainly distributed among transcription factors, hydrolases, enzyme modulators. In addition, the number of proteins associated with energy and lipid metabolism was also high, including fatty acid desaturase, lipase, lipoxygenase, regulator of metabolic enzymes, etc. This result was consistent with the GO analysis and DEGs identification analysis, suggesting that lipid metabolism and the immune system may play an important role in the mechanism of progesterone resistance.

KEGG pathway enrichment of the 821 DEGs was conducted using the Panther database. Twenty-nine pathways were enriched with a criterion of p < 0.05 (Table 2, Fig. 4b). Complement and coagulation cascades, signaling pathways regulating pluripotency of stem cells and Staphylococcus aureus infection pathway were highly enriched in upregulated genes. Transcriptional misregulation in cancer, Rap1 signaling pathway and cancer related pathways were the most obvious pathways in downregulated genes. Among the 29 pathways, 11 pathways were involved in the immune system. Five pathways were related to extracellular environment and epithelia mesenchymal transition (EMT). This analysis suggested that extracellular environment, EMT and immune system may be critical in the development of progesterone resistance.

**Table 2:** Top 5 KEGG pathway enrichment of up-regulated and down-regulated DEGs sorted by p-value and count of hit-genes

Term	Description	p-value	Count
Up-regulated			
hsa04610	Complement and coagulation cascades	1.40E−04	9
hsa04550	Signaling pathways regulating pluripotency of stem cells	0.001138056	11
hsa05134	Legionellosis	0.001227629	7
hsa05150	Staphylococcus aureus infection	0.001227629	7
hsa05133	Pertussis	0.006513616	7
Down-regulated			
hsa05202	Transcriptional misregulation in cancer	0.003670966	9
hsa04015	Rap1 signaling pathway	0.004207876	10
hsa05200	Pathways in cancer	0.014545204	13
hsa04918	Thyroid hormone synthesis	0.020795767	5
hsa05146	Amoebiasis	0.021449539	6

### PGR coexpression analysis and validation

**Fig. 5:**
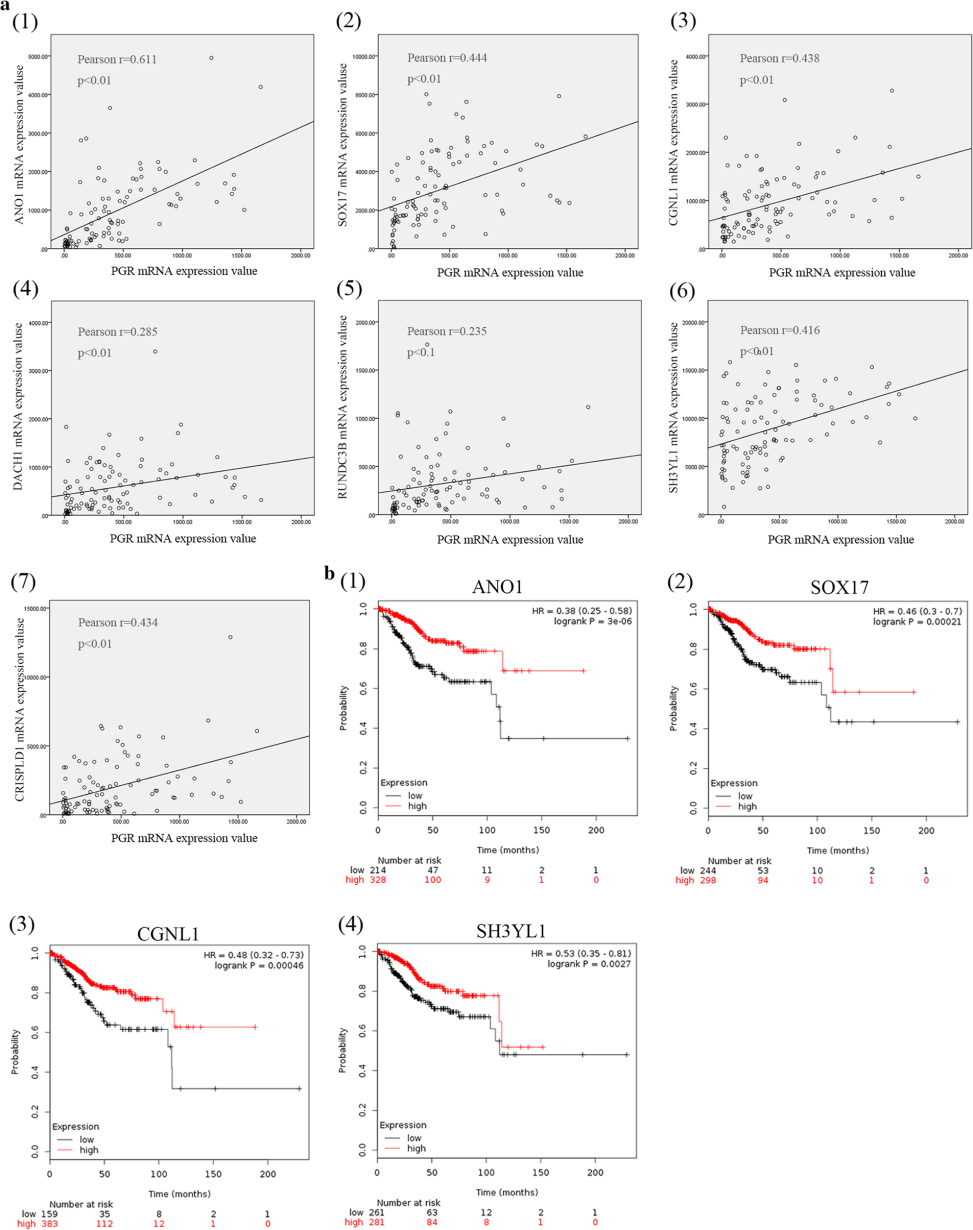
Correlation analysis and Kaplan–Meier survival curves of PGR co-expressed genes. **a** Correlation analysis using GSE17025 data. (1)–(7) were 7 positively PGR correlated genes (ANO1, SOX17, CGNL1, DACH1, RUNDC3B, SH3YL1, CRISPLD1) that were also down-regulated in IshikawaPR cell. **b** Kaplan–Meier survival statistics analysis revealed that 4 (ANO1, SOX17, CGNL1, SH3YL1) out of 7 target genes were dramatically correlated with prognosis

**Fig. 6:**
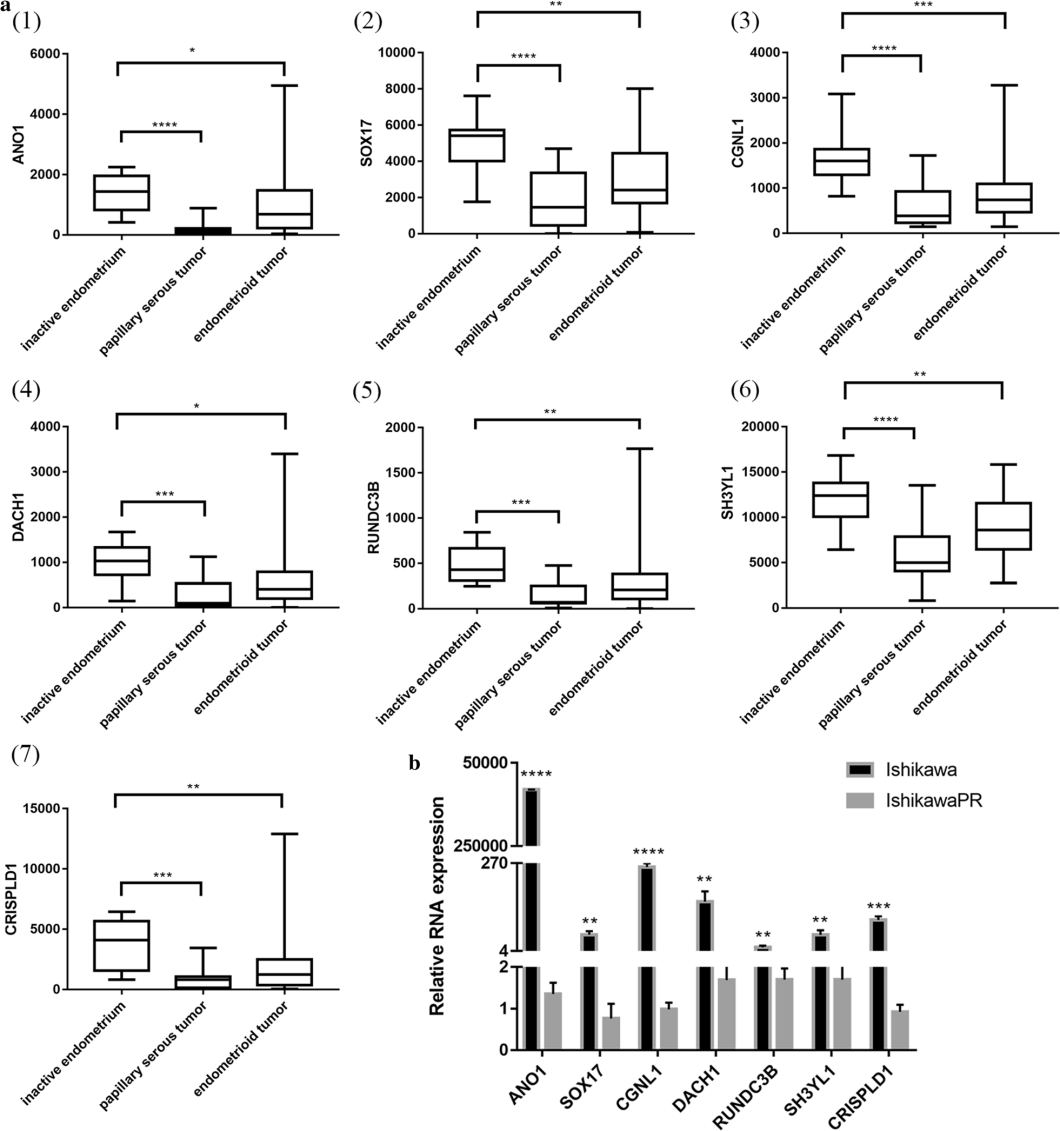
Validation of 7 PGR co-expressed genes. **a** Validation by using GSE17027 91 which contains 91 stage I endometrial cancer patients and 12 postmenopausal healthy women’s endometrium. (1)–(7) were 7 genes (ANO1, SOX17, CGNL1, DACH1, RUNDC3B, SH3YL1, CRISPLD1) that were both significantly down-regulated in papillary serous tumor and endometrioid tumor compared with inactive endometrium. **b** RT-PCR validation of 7 PGR co-expressed genes in IshikawaPR cell and Ishikawa cell

PGR coexpression genes within endometrial cancer were identified from cBioPortal which is based on the TCGA database, including 549 endometrial cancer tissues. In total, 859 PGR coexpressed genes were selected with |Pearson score| > 0.3 and |Spearman score| > 0.3 as criteria (Additional file 5: Table S5). In total, 36 genes overlapped between DEGs and PGR coexpressed genes (Additional file 6: Table S6), including 32 PGR positively correlated and 4 negatively correlated genes. Regarding these 36 genes, 25 genes were downregulated in IshikawaPR cell and positively correlated with PGR, and 2 genes were upregulated in IshikawaPR cells and negatively correlated with PGR. To validate these target genes, GEO dataset GSE17025 [19], which included 91 Stage I endometrial cancer patients and 12 postmenopausal healthy female tissues, was used to calculate correlation coefficient (Fig. 5). Fifteen genes were dramatically correlated with PGR expression, including 14 PGR positively correlated genes (ANO1, PLCB1, NPAS3, SH3YL1, SOX17, SLC40A1, CCDC146, RUNDC3B, SEMA3D, CRISPLD1, CGNL1, CDS1, XIST and DACH1) and 1 PGR negatively correlated gene (SLCO3A1). We further screened their expression patterns among these 15 genes. As shown in Fig. 6a and Table 3, ANO1, SOX17, CGNL1, DACH1, RUNDC3B, SH3YL1 and CRISPLD1 were significantly downregulated both in papillary serous tumors and endometrioid tumor.

**Table 3:** Distinguishing and validation of 7 PGR co-expressed genes among DEGs

Gene symbol	PGR co-expression (548 EC tissues)		Microarray analysis		GSE17025		Survival curve (542 EC tissues)	
					Pearson score			
	Pearson score	Spearman score	LogFC	−Log(p-value)	r	p-value	HR	LogRank p-value
ANO1	0.66	0.72	− 10.78	8.69	0.611	< 0.01	0.38	3.00E−06
CGNL1	0.39	0.45	− 7.19	3.38	0.438	< 0.01	0.48	0.00046
DACH1	0.33	0.39	− 6.67	4.60	0.285	< 0.01	0.79	0.27
SH3YL1	0.58	0.54	− 4.40	6.41	0.416	< 0.01	0.53	0.0027
RUNDC3B	0.43	0.46	− 4.23	4.64	0.235	< 0.1	0.73	0.21
CRISPLD1	0.39	0.39	− 4.09	4.42	0.434	< 0.01	0.64	0.038
SOX17	0.46	0.59	− 4.03	5.83	0.444	< 0.01	0.46	0.00021

PGR co-expression showed correlation analysis among target genes and PGR using Cbioportal website. The third column shows the result of microarray analysis between Ishikawa and IshikawaPR cell. Negative means they were down-regulated in IshikawaPR cell. The fourth column is the PGR correlation analysis of target genes using GSE17025 dataset. Survival curve was performed using Kaplan–Meier plotter. FC, fold change

To determine the clinical significance of 7 target genes, Kaplan–Meier survival statistics were generated for a large cohort of endometrial cancer. In total, data from 542 endometrial cancer patients were interrogated and hazard ratios (HR) and p-values for statistical significance were determined. The data are summarized in Table 3. Interestingly, low expression of the 7 target genes correlated with poor prognosis especially ANO1, SOX17, CGNL1 and SH3YL1 (Fig. 5b, Additional file 7: Figure S1). These 7 target genes should be further studied to explore their association with PGR expression, which might expose the mechanism of progesterone resistance as well as downregulation of PR during the development of endometrial cancer.

### Protein-protein interaction network (PPI) and modular analysis

**Fig. 7:**
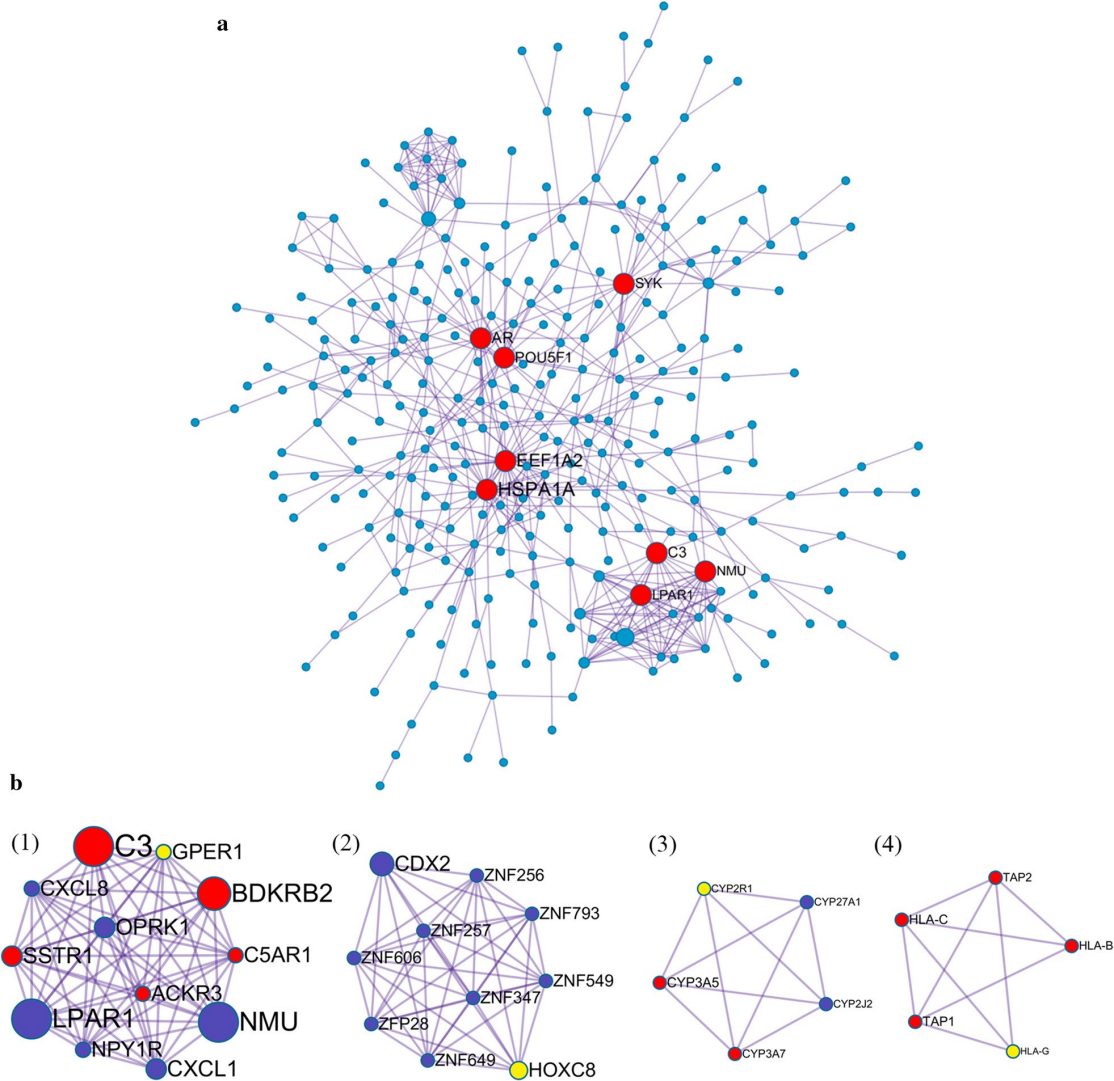
Protein-protein interaction (PPI) network and identification of MCODEs. **a** PPI network of 821 DEGs and 8 hub genes. Red represented hub genes (degree ≥ 15 as cut-off criterion). Font size of gene symbol determined by degree of connectivity. The width of the edge was determined by the combined score of the PPI relationship. Hub genes were HSPA1A, EEF1A2, AR, POU5F1, C3, SYK, LPAR1, NMU. **b** 4 MCODEs identification in the PPI network with MCODE score ≥ 4. The label size was determined according to the degree of connectivity. Yellow represented seed nodes. Red nodes were up-regulated genes and blue nodes were down-regulated genes in IshikawaPR cell

The protein–protein interaction (PPI) network was generated using Metascape and visualized with Cytoscape 3.5.1. Proteins with degree > 1 were selected. In total, 352 nodes (42.9% of all 821 DEGs) and 592 PPI relationships were obtained (Fig. 7). Eight genes with a degree of connectivity > 15 were defined as hub genes for progesterone resistance (Table 4, Fig. 7a). According to the degree rank, the eight hub genes included HSPA1A, EEF1A2, AR, POU5F1, C3, SYK, LPAR1 and NMU. The 8 hub genes interact directly with 112 DEGs. As the most intensive hub gene, HSPA1A interacts with 15 upregulated and 15 downregulated genes. In addition, these hub genes could interact with each other. AR could interact with three hub genes (HSPA1A, EEF1A2, SYK), HSPA1A, EEF1A2, C3, LPAR1 and NMU could interact with other 2 hub genes. These results suggest that these hub genes might play an important role in progesterone resistance and should be further studied.

**Table 4:** 8 hub genes (degree ≥ 15) selected from PPI network according to degree

Gene symbol	Eccentricity	Closeness	Betweenness	Degree	Stress
HSPA1A	0.1667	0.0011	26,447.0664	30.0	65,244.0
EEF1A2	0.1429	0.0010	13,663.9556	26.0	38,054.0
AR	0.1667	0.0011	15,814.7167	20.0	49,698.0
POU5F1	0.1429	0.0010	14,493.2442	19.0	42,006.0
C3	0.1429	0.0009	5703.5021	18.0	14,084.0
SYK	0.1429	0.0010	12,162.3216	16.0	34,430.0
LPAR1	0.1250	0.0008	996.2218	15.0	3726.0
NMU	0.1250	0.0008	1251.3925	15.0	3778.0

Ten densely connected regions in the networks were identified using the MCODE clustering algorithm by Metascape [20]. Four most significant MCODEs were extracted when the node score cut-off was 0.2 and K-core was 2 (Fig. 7b). GO enrichment analysis of the 4 MCODEs-related genes showed that these genes were mainly associated with the following biological processes terms: antigen processing and presentation, immune response, G-protein coupled receptor signaling pathway and chemotaxis. Regarding molecular function terms, these genes are mainly enriched in steroid hydroxylase activity, heme binding, oxidoreductase activity, aromatase activity and peptide antigen binding. Regarding cellular component terms, the top terms were MHC class I protein complex, intracellular and integral component of plasma membrane. The top 15 GO enrichment terms are presented in Table 5. KEGG pathway enrichment results showed that these genes were significantly enriched in antigen processing and presentation, phagosome, graft-versus-host disease, allograft rejection, and some microbials infection (Table 6). These results were consistent with the GO and KEGG pathway enrichment analysis of DEGs, suggesting that the immune system and lipid metabolism play an extremely important role in progesterone resistance and should be further studied.

**Table 5:** Top 15 GO enrichments of the 4 MCODEs related genes

GO term	Description	−Log10(p)
GO:0002474	Antigen processing and presentation of peptide antigen via MHC class I	6.60
GO:0006955	Immune response	3.98
GO:0002480	Antigen processing and presentation of exogenous peptide antigen via MHC class I, TAP-independent	3.93
GO:0006954	Inflammatory response	3.22
GO:0090023	Positive regulation of neutrophil chemotaxis	3.13
GO:0007218	Neuropeptide signaling pathway	3.08
GO:0007186	G-protein coupled receptor signaling pathway	2.98
GO:000693	Chemotaxis	2.84
GO:0042612	MHC class I protein complex	3.82
GO:0005622	Intracellular	3.57
GO:0005887	Integral component of plasma membrane	3.38
GO:0031090	Organelle membrane	3.37
GO:0071556	Integral component of lumenal side of endoplasmic reticulum membrane	2.96
GO:0008395	Steroid hydroxylase activity	4.85
GO:0020037	Heme binding	3.96

−Log10(p): −Log10(p-value)

**Table 6:** Top 10 KEGG pathway enrichment of the 4 MCODEs related genes

KEGG pathway term	Count	−Log(p-value)
hsa04612:Antigen processing and presentation	5	4.241909
hsa04145:Phagosome	6	4.228601
hsa05168:Herpes simplex infection	6	3.858941
hsa04080:Neuroactive ligand-receptor interaction	6	3.025758
hsa05332:Graft-versus-host disease	3	2.399844
hsa05330:Allograft rejection	3	2.302013
hsa04940:Type I diabetes mellitus	3	2.194244
hsa05320:Autoimmune thyroid disease	3	2.014217
hsa05134:Legionellosis	3	1.982622
hsa05416:Viral myocarditis	3	1.93748

Count: the number of MCODEs related genes contained in the pathway

## Discussion

Endometrial carcinoma (EC) is the most common gynecologic malignancy. De novo or acquired progesterone resistance is a major clinical problem. Our study is the first comprehensive investigation to explore the mechanism of progesterone resistance by microarray analysis. We developed an endometrial cancer cell subline that exhibits stabilized resistance to MPA and performed microarray analysis to identify DEGs. In total, 821 DEGs, 7 PGR coexpressed genes, 8 hub genes and 4 MCODEs were obtained. Using GO and KEGG pathway enrichment analysis, protein class analysis, PGR coexpression identification and validation, PPI and modular analysis of DEGs, we further explored the following possible mechanisms involved in progesterone resistance.

### Identification of predictive biomarkers for progesterone treatment response

Previous research suggested several prognostic markers for response to progesterone treatment, including progesterone receptor (PR) [21], survivin [22], Fas/FasL [23], Nrf2 and AKR1C1 [24], DUSP6 [25] and DHCR24 [26]. These findings were all based on exiting research and lack a systematic view of the molecular network of progesterone resistance.

Our study is based on microarray analysis of 27,831 expressed genes, representing a more comprehensive and integrated exploration of the internal mechanism. Predictive biomarkers could identify patients who would benefit from the treatment. In total, 821 DEGs were identified from microarray analysis, including 760 coding RNA, indicating that these gene coding proteins might serve as protein biomarkers. By detecting these predictive biomarkers before treatment, patients who will be insensitive to progesterone can be screened, and can be recommended for other treatments, avoiding disease progression. In addition, the development of targeted drugs against drug resistance will increase the sensitivity to progesterone and help to provide benefit from conservative treatment. Additional validation studies are needed to verify the biomarkers value and optimal filter conditions.

### Potential mechanistic hypotheses of PR dysregulation

The presence of the progesterone receptor (PR) is the precondition for progesterone response and PR is a predictive marker for response of progesterone [21]. Progesterone binds to its receptor PR-A and PR-B, subsequently inhibiting tumor growth and promoting tumor apoptosis by regulating downstream genes. Constant stimulation of progesterone reduced the expression of PGR [10] and promoted the development of drug resistance. Thus, downregulation of PR especially PRB must be involved in progesterone resistance. However, the molecular mechanism of PGR dysfunction remains unclear.

A study defined the genome-wide PR cistrome in the murine uterus using Chip-seq to identify novel PR target gene in circadian rhythm reveled that Sox17 is a novel mediator of progesterone signaling in normal endometrium [27]. After acute progesterone treatment, Sox17 is upregulated at both the mRNA and protein levels. In our study, PGR coexpression analysis showed that Sox17 was negatively correlated with PGR and downregulated in IshikawaPR cell. These results suggest that when resistance occurred, Sox17 was downregulated rather than upregulated in normal endometrium treated with progesterone. Kaplan–Meier survival statistics revealed that its higher expression in endometrial cancer patients is associated with better survival. Targeting Sox17 may represent a new approach to reverse resistance.

In a recent study, Huizhe Wu [28] reveals that the calcium-activated chloride channel Ano1 promotes cell proliferation in ER-positive, PR-positive, and HER2-negative breast cancer cell, but inhibits cell growth in ER-negative, PR-negative, and HER2-negative breast cancer cells. These results suggest that Ano1 may differentially regulate cell proliferation depending on the status of steroid receptors. In breast cancer patients treated with tamoxifen, overexpression of the selective estrogen-receptor modulator, Ano1 is associated with good prognosis in PR-positive patients [29]. Similar to our study, Ano1 was downregulated in IshikawaPR cell, which is a PR-negative cell. Positive correlation was noted between Ano1 and PGR. High Ano1 expression is essential for survival and its expression was altered in papillary serous tumor and endometrioid tumor compared with healthy endometrium. Strategies to activate Ano1 are needed to promote the response to progesterone.

PR must first recruit coregulators with intrinsic histone and DNA-modifying activities and then regulate target gene. Many coregulators of hormonal receptor have been identified and Src homology 3 domain containing, Ysc84-like 1 (SH3YL1) is a novel coregulator that bind to the polyproline domain of androgen receptor (AR) [30] in prostate cancer cell. In our study, SH3YL1 was downregulated in resistant cell and positively correlated with PGR. SH3YL1 expression was considerably in endometrial cancer tissues compared with inactive endometrium. Whether SH3YL1 is also the coregulator of PR remains to be investigated.

Other PGR coexpressed genes and their encoded proteins were all potential PR targets and should be further studied. Nrf2 (Nuclear factor erythroid related factor 2), overexpression of which results in progesterone resistance in endometrial cancer cells [24], binds to the SLC40A1 promoter and transcriptionally suppressed SLC40A1 expression [31]. SLC40A1 subsequently promotes Nrf2 expression. In addition, SLC40A1 is associated with cisplatin resistance in ovarian cancer. Consistent with our results, Nrf2 was upregulated and SLC40A1 was downregulated in IshikawaPR cells. Interestingly, SLC40A1 was positively correlated with PGR and exhibited reduced expression in endometrial cancer tissues than normal endometrium, indicating a potential relationship between SLC40A1 and PGR dysfunction as well as a mechanism of endometrial cancer proliferation.

Furthermore, NPAS3, CCDC146, RUNDC3B, SEMA3D, CRISPLD1, CGNL1 and CDS1 were all negatively correlated with PGR and have not been thoroughly studied. Thus, further understanding their function within progesterone resistance is required.

### Immune system and inflammatory response may be associated with progesterone resistance

This study first found that the immune system and inflammatory response are potentially associated with the development of progesterone resistance. Immunomodulation in endometrial cancer and chemoresistance have been studied for years but have never been studied in the context of progesterone resistance in endometrial cancer. The immune system has pro- and anti-tumorigenic functions in the endometrium. By forming an integral mucosal immune system, normal endometrial epithelial cells could secrete defensin [32], which exhibits immediate antimicrobial function and the ability to active the adaptive immune system by attracting T cells and dendritic cells (DC) [33]. Endometrial epithelia cells are antigen-presenting cells that express major histocompatibility complex (MHC) and non-classical MHC class I molecules and human leukocyte antigen G (HLA-G) is down regulated in endometrial cancer [34]. In our study, HLA-G was downregulated in IshikawaPR cell and a MCODEs gene. HLA-G maybe offer protective function to avoid NK cell lysis.

Hormones influence the inflammatory environment. Estrogen increases the expression of inflammatory cytokines released from murine and uterine cells but progesterone decreases their production [35]. A previous study demonstrated that the inflammatory responses is increased in mouse uterus lacking PGR, and infiltration of leukocytes and extensive tissue remodeling also increase [36]. Alterations in steroid receptors expression leads to an increase in estrogen to progesterone signaling, subsequently increasing inflammatory mediators and promoting tumor growth [37]. Many inflammatory response and immune system related GO terms were significantly enriched in our study. Within the hub genes, six of the top 20 GO enrichment terms of MCODEs genes were involved in immune response and immune systems, especially antigen process and presentation of peptide antigens. Genes involved in these terms included TAP1 (ATP-binding cassette) and TAP2, which are transporters associated with antigen processing and are major contributors to the multidrug resistance phenotype [38]. In addition, CXCL1 and CXCL8 are chemokines that are elevated in endometrial adenocarcinoma [39, 40]. However in progesterone resistant cells, these genes are downregulated, requiring further study.

Another potential mechanism of progesterone resistance involves the G protein-coupled estrogen receptor 1 (GPER1), which mediates immunomodulation. GPER1 was upregulated in progesterone resistant cell in our study and is also a MCODEs genes. Given that estrogen anti-inflammatory activity is mediated by estrogen receptor ERα36 and GPR30/GPER1 [41], the increase in GPER1 expression in progesterone-resistant cells may hasten the inflammatory response and promote the proliferation effect by estrogen. Our study provides supportive evidence for the novel theory that the immune system and inflammatory response play vital role in progesterone resistance of endometrial cancer. These findings may provide new strategies to block the immune response to reverse the progesterone resistance in the future.

### The activation of lipid metabolism is vital in progesterone resistance

In cancer cells, previous studies declare that metabolism alterations including aerobic glycolysis and de novo lipid biosynthesis promote the transformation of cells from a noncancerous to cancerous state [42]. Given that fatty acids provide twice as much ATP as glucose and are preferred nutrients for storage, they serve as an energy source when the requirement for ATP production increases in cancer cells [43]. Several studies have demonstrated the vital role of fatty acid metabolism in endometrial cancer progression. We previously reported that SREBP1 promoted fatty acid metabolism by regulating the transcription of FASN and played an important role in the tumorigenesis of endometrial cancer [44]. In addition, in endometrial cancer cell, SREBP1/FASN was suppressed during the proliferation suppression and apoptosis induced by progesterone [45]. In this study, the expression of genes involved in fatty acid biosynthetic processes, lipid metabolic processes, lipid translocation and regulation of lipid metabolic process related genes were all increased. However, genes involved in the lipid biosynthetic process were decreased. Consistent with KEGG pathway analysis, elevation of fatty acid synthesis and lipid metabolism were extremely important in progesterone resistance.

## Conclusions

We offer a comprehensive analysis of gene expression profile in progesterone resistant endometrial cancer cell and raise some novel, potential mechanistic hypotheses of progesterone resistance. We identified 821 DEGs, 7 PGR coexpressed genes, 8 hub genes and 4 MCODEs that are significantly enriched in immune system, inflammatory response, lipid metabolism and extracellular environment. These findings provide comprehensive information for further study how to promote the response of progesterone treatment in endometrial carcinoma.

## Additional files

**Additional file 1: Table S1.** 821 differential expressed genes (DEGs) were identified from microarray profile.

**Additional file 2: Table S2.** The information of primers used in this study.

**Additional file 3: Table S3.** Top 30 DEGs sorted by |log2foldchange|.

**Additional file 4: Table S4.** Microarray analysis result of genes related to lipid metabolic and biosynthetic process in IshikawaPR and Ishikawa cell.

**Additional file 5: Table S5.** PGR co-expression genes within endometrial cancer were identified form cBioPortal. 859 significantly PGR correlated genes were selected.

**Additional file 6: Table S6.** Distinguishing and validation of 36 genes that were overlapping of PGR co-expression genes and DEGs.

**Additional file 7: Figure S1.** The survival curves of 7 PGR co-expressed genes comparing the patients with high (red) and low (black) expression in endometrial cancer.

## Abbreviations

MPA: medroxyprogesterone acetate
DEGs: differential expressed genes
PPI: protein–protein interaction network
MCODEs: molecular complex detections
AEH: endometrial hyperplasia
EC: endometrial cancer
PGR: progesterone receptor
