# Supplementary material for: Comprehensive bioinformatics analysis of acquired progesterone resistance in endometrial cancer cell line
Source: J Transl Med. 2019 Feb 27;17:58. doi: 10.1186/s12967-019-1814-6 (PMC6391799; doi:10.1186/s12967-019-1814-6)
Supplement: Supplementary file 1 — Additional file 1: Table S1. 821 differential expressed genes (DEGs) were identified from microarray profile. [file 12967_2019_1814_MOESM1_ESM.docx]

**Additional Table S1**. 821 differential expressed genes (DEGs) were identified from microarray profile, including 453 up-regulated genes and 368 down-regulated genes in IshikawaPR cell compared to Ishikawa cell. The genes list were ranked according to Fold Change.

| **DEGs** | **Geny symbol** |
| --- | --- |
| up-regulated genes (453) | VIM,EFEMP1,WISP2,CPS1,SPINK13,NPTX1,MT1M,TM4SF1,LINC00473,LPL,WNT5A,NNMT,LY6K,CSGALNACT1,CDH13,SSTR1,SRGN,AHRR,FAM179A,CARD16,CT83,DAB2,TBX3,CARD6,KYNU,ANXA8L1,CASP1,TAP2,COL16A1,C4BPB,HOXC9,ZNF469,SLC16A6,GAGE7,KCNQ1,SLCO3A1,PSCA,SLC17A9,HRK,KIF26A,GRID1,NR2F2,CGA,S100P,IFI16,KRT14,MGLL,BDKRB2,LAMA1,CLMP,OLFM1,CRIP1,HLA-B,MAPK4,FZD10,FOXD1,GPR133,HES7,PTGES,PTGS1,LINC01021,TBX1,PIP5K1B,HLA-C,ME3,PSORS1C3,ITPR1,RASSF2,CRB1,LINC00847,NOG,MGAT5B,PARP14,HBG1,PLEKHA6,LINC00526,NOTUM,LCN15,KRT17,SKIDA1,GAL3ST1,CFH,SECTM1,SCARA3,PDE2A,FOXL2NB,SYT12,STAT6,APOL6,ADAMTS8,HOXC6,EEF1A2,FOLR3,FGF2,DUSP1,ODF3L1,ABCA13,NEBL,NKX2-1,DLX4,MMP24,PLIN2,PSMB8,VWA5A,RNF182,TFAP2A,LINC00667,FOXE1,SYPL2,MMP7,PTPRM,KLHL29,SCARA5,LOC400768,TVP23A,SVEP1,NRP1,CLU,DNER,HRASLS2,HOXB3,SLIT1,IFIT5,PLAC8,GAS1,MVP,DNMT3L,FA2H,PDE4D,GDA,C5AR1,FBXL16,FOLR1,EMB,ASTN1,LOC283713,CYP3A5,KCTD15,SP100,HOXB7,HOXC13,MEIS2,CARD17,PDE3A,ABLIM2,LOC643486,HBE1,DKK1,XAGE2,IGFBP7,FHL2,MUC1,FLJ32255,SPON2,CPNE8,C8orf4,CASP4,TRIM29,AUTS2,HOXC10,SVOPL,PAPPA,INSL4,HOXB2,HOXB6,C11orf86,DOCK8,CACNA2D3,GJC1,LINC00942,HOXC4,PTPRD,DPPA2,RBP4,ST3GAL1,SLC51B,NR4A1,OAS1,DKFZp434J0226,CFHR3,LOC100268168,TNS4,LIFR,TPM2,TMTC1,SCIN,ST3GAL4-AS1,HKDC1,IL18,RFTN1,GBP3,UNC5B,CECR7,EHD2,ANO4,HSPA1A,MAMDC2,LOC100049716,SOX18,EVPLL,LINC00312,NCF2,RBM43,PAGE1,RHBDL3,POU5F1,ANO2,LINC00842,LOC100128242,WHAMMP3,LOC645638,UBD,SP140,HES5,TRIML2,PIEZO2,GABRB3,POF1B,H19,ALPP,ACKR3,MYLK,NUDT9P1,FTMT,OLAH,LOC375196,GALNTL6,HMOX1,NEFH,RASIP1,PARP9,CASP7,LINC00525,SH3PXD2A,C1R,PTPRU,TCAM1P,C1RL,TDRD9,HIVEP3,NRIP3,LOC284080,GUCY2EP,INHBB,OR51I1,FAM111A,SLMAP,ITGB2,LINC-PINT,TMEM101,ATP6V0A4,RBM24,CDYL2,BST2,CSF2RA,PNMA2,SLC6A8,ZNF503,AQP3,SEC14L4,C1S,LOC100287290,DMKN,ACOT1,FOXF2,LOC100132356,CEACAM7,HORMAD1,COPZ2,TNFRSF14,KRT16P2,LINC01124,SLC2A5,FXYD3,GNAO1,OTUD7A,LIPH,FBN1,NPR3,DOCK5,APC2,GATA6,DLG3,FENDRR,PSORS1C1,BIRC3,FOSL2,AR,COL12A1,ALPPL2,C1QTNF6,MGC24103,LOC101928837,LFNG,PRSS23,DRGX,ANXA2R,LOC100506688,SYCP2,HOXB5,KRT80,ENOX1,OR51B4,ADARB1,CACNA1C,HOXC8,NXNL2,FRMD3,PERM1,PCSK1,TAP1,ZNF503-AS2,PTRF,LZTS1,PSAPL1,NDRG1,HNMT,LOC284561,GDF6,ZBTB20,SYBU,OR51E2,SPINK5,C10orf11,TUG1,HOXA11-AS,OPN3,TNN,MGC50722,ARSG,KLHL3,DTX3L,CD55,RAB26,GATA5,VAV2,CASP5,COL15A1,KLHL35,C16orf45,JAK1,MYPN,STEAP4,LAMB3,TRABD2A,PDLIM3,GLI3,VGLL3,AREG,AATK,MDGA1,SLC45A4,GAGE1,GATS,EPHA1-AS1,SYNC,OLFML2A,RYR1,AHNAK,GPER1,C1RL-AS1,ERAP1,BEGAIN,C2orf90,IRAK3,MFAP5,LINC01270,DOK7,AOX1,KIAA0319,LOC285696,SP110,ASS1,C3,BMP2,HOTAIR,PIK3C2B,LOC388282,TPPP,STOM,CYP3A7,NPDC1,HECW2,LOC284219,MIR100HG,TIMP2,CRLF1,GABRE,FAM122C,LINC00324,EDN2,ABLIM3,KAZALD1,ACAA2,IL20RB,LINC00899,MYLK3,RORA,GRIN2D,FAM46A,FAM111B,FTCDNL1,KCNN4,ATP8B3,PDE8B,TLE4,PMAIP1,NR4A3,HLA-G,MT1E,ALOX5,LOC442132,HIST1H1A,NALCN,LOC100127909,LOC284570,PDGFRB,S100A4,FOXL2,GMPR,DFNA5,PRR5L,HSPB8,EDIL3,HSPB3,CSPG4,FOXL1,SLC22A3,ZNF655,SSR4P1,BAHCC1,STAT5B,PDLIM2,LOC101928841,H6PD,COL4A3,KIAA0825,DIO3OS,STAT5A,ABTB1,ZNF556,AKT3,FOXF1,DMBT1,AKR1C4,GPR1,MYOF,IGFBP6,CFB,NNT,TBXA2R,AVPI1,MSLN, |
| down-regulated genes (368) | SPON1,ANO1,CLDN3,SPRY1,KCNE3,FRG2C,PCDH7,HMGA2,SHISA3,DUSP6,NPAS3,BCAT1,FRG2,EMX2,C3orf14,CHGA,XIST,KIF1A,SALL1,LAD1,DDX43,TMEM30B,HRASLS,ZSCAN18,XLOC_014512,FRG2B,AFAP1L2,NMU,BEX4,VSNL1,TMSB15B,ZNF850,GRHL2,SOBP,EPN3,LOC101927418,IGFBP2,DDIT4L,ZNF730,LONRF2,GJA1,COCH,SLC15A1,SPP1,THSD7A,AMIGO2,ZNF667-AS1,BAGE,KLHL14,VCX2,ZNF793,PNMAL1,PTPN13,BEX1,ZNF204P,ZNF256,CGNL1,LOC100130899,ADAP2,CCDC3,RBP7,SMOC2,FHOD3,CNTNAP2,CCDC149,CDH18,C2orf54,MAP9,ZNF518B,STK33,LOC100129055,PZP,FGF12,ZNF682,SLAIN1,SLC10A4,RPS6KA2,ZNF649,LEMD1,DACH1,DDX26B,ETV5,CYB5R2,WFDC2,KRT19,SALL2,SPRY2,SEMA3D,PROM1,ZNF671,DLX6,TLR2,TRIM43,TSTD1,SPINT1,MYEF2,PTPN20B,ADAMTS18,OGDHL,ZNF681,BEND7,ST6GALNAC1,ID4,FAM89A,SLC1A3,FABP6,GALC,PCDHB5,SRSF12,CMTM7,FRAS1,ERICH5,SPRY4,AS3MT,GPR98,KRT19P2,BEND5,LMF1,ZNF347,ZNF711,ZNF608,BSPRY,CDX2,CSAG1,PXMP4,ZNF577,PPP1R1B,DNALI1,KDF1,NAP1L3,NGEF,LOC101060810,MKX,MDK,CPVL,ANKRD6,CSAG4,SLIT2,LRCH2,RD3,PCDH20,ABHD12,PDGFC,SULT1C2,SLC40A1,LRRTM4,SLC16A10,TMOD1,ZNF549,IL1R2,ZNF880,PAX8,PTH2R,LRAT,FAM117A,AGR2,SEMA3A,SPINT2,TCEAL6,SH2B3,PGF,RAB25,GNA14,PRDM13,EPHB3,SOWAHA,MSX1,CPE,JAG1,GUCY1B3,RAPGEF3,LINC00839,TRIM49C,CDH1,MAGEC2,SPESP1,NSG1,EPCAM,RLN2,LYPD6B,MIR181A1HG,CABYR,FAM221A,TCEAL5,SOD3,ANGPTL1,B4GALT6,LPAR1,LOC729680,ZC4H2,CYP2R1,ADAMTS3,SMO,MAATS1,EMX2OS,LGR5,MXRA5,SCARNA13,UBASH3B,KIAA2022,IL17RD,ACSS3,FGF18,ALDH1L1,MOXD1,PBDC1,DLX5,TET1,KRT23,ATP1A2,BEX5,ZNF415,ELOVL4,CXCL1,LOC100129098,CDH6,SLC51A,ARMC4,ANO5,LOC100129322,CCNYL2,TMEM98,ALDH1A1,TRIM2,GPX7,DLC1,MFSD2A,SAGE1,RBM11,MCOLN3,LINC01127,LOC285300,CGN,FKBP1A,ITGB3,SBK1,GPX3,LPHN2,C6orf52,ZNF606,FAM65C,ID2,FBXO36,OOEP,SOSTDC1,ANKRD34B,NCMAP,MEX3A,NAP1L2,PRINS,NPY1R,SMIM22,HGD,SHROOM2,GRIA4,TRIL,TMEM45B,MARVELD3,RLN1,BLNK,CA8,FAM159B,SUSD5,PACSIN1,MGAT3,ENPP5,INPP4B,RAB17,STK31,NFE2L3,RPL22L1,ZBTB8B,ELOVL6,ZNF257,ATP8B1,LOC100129940,TNFRSF19,TNFSF10,NID1,ZNF773,PRKAR2B,CYYR1-AS1,VWA1,CCDC146,KCNRG,CAPS2,C2orf88,FUT2,HOOK1,OPRK1,SH3YL1,DHRS2,RASL11B,CLIC6,STYK1,CYS1,PRSS8,PCDH9,KCTD8,CHST1,BEX2,ZFP28,ZNF43,C8orf48,CDS1,OVOL1,TNFRSF11B,DYRK2,SOX9,DPY19L2P1,PRICKLE1,STEAP1,SYK,SAG,MCOLN2,SLC43A2,DUSP26,NFE2,PLCB1,MARCKSL1,GPC4,MERTK,TSPAN5,RUNDC3B,FLJ13744,LAMC2,CXCL8,POTEI,GADD45G,HES6,BMX,KBTBD8,WNT3A,FGF19,RAB15,PALM,TBX2,XG,BEND4,MEIS1,PLXNA4,PRR15L,AGR3,CLCN4,SLC22A23,STC1,S100A14,FBP1,ZNF215,SLC16A14,CRISPLD1,RBP1,CLEC11A,SLC9A2,SLC26A7,CLIC2,CLDN1,RASSF4,CYP2J2,ICA1,CYP27A1,C1orf168,SOX17,PCDHB6,ADAMTS17,FZD5,CCNB3,MUM1L1, |
