# Supplementary material for: Comprehensive bioinformatics analysis of acquired progesterone resistance in endometrial cancer cell line
Source: J Transl Med. 2019 Feb 27;17:58. doi: 10.1186/s12967-019-1814-6 (PMC6391799; doi:10.1186/s12967-019-1814-6)
Supplement: Supplementary file 2 — Additional file 2: Table S2. The information of primers used in this study. [file 12967_2019_1814_MOESM2_ESM.docx]

**Additional Table S2**. The information of primers used in this study.

| **Gene name** | **Primer sequence** | |
| --- | --- | --- |
| ANO1 | Forward | CATCGAGGACATCGGCTACC |
|  | Reverse | TTTTAGTGTCCTCGTCCCGC |
| PLCB1 | Forward | GAAGCGCAAAGTAAACGGCA |
|  | Reverse | CTGCAGCTTGGGCTTTTCAT |
| NPAS3 | Forward | CCTTCCAGGCGAGAACGAAT |
|  | Reverse | CTTGTCGAGCTGGCTGGTAA |
| SH3YL1 | Forward | ACTCTTTGCAGGCGTGTCTT |
|  | Reverse | AGATCTTCGGCTTGAGCAGG |
| SOX17 | Forward | CCCTTCACGTGTACTACGGC |
|  | Reverse | TGTTCAAATTCCGTGCGGTC |
| SLC40A1 | Forward | GATCCTTGGCCGACTACCTG |
|  | Reverse | CACATCCGATCTCCCCAAGT |
| CCDC146 | Forward | TGGCAGCGTTAAAAGCCAAG |
|  | Reverse | TGGAGACCTCCGTGGAGAAT |
| RUNDC3B | Forward | TGGATGGCAGTTTTCCTGCT |
|  | Reverse | ACCACTGCTTCCCAAAGTCC |
| SEMA3D | Forward | GCCAGAAGCCAAGATTTTCAC |
|  | Reverse | ATGCTCCAGTTCCACACACA |
| CRISPLD1 | Forward | CTGTTCTGCTTGCCCACCTA |
|  | Reverse | CAGCCAGCAGGACATTCGTA |
| CGNL1 | Forward | GCAATTCCCAGCCTACCAGT |
|  | Reverse | TGACGTGGGAGTTGTGGATG |
| CDS1 | Forward | TGGGAAGGATTCATTGGTGGTT |
|  | Reverse | GGAAAGGGTACAAGCTCACT |
| XIST | Forward | GTTCTGTCGCAGTGTTCAAGTG |
|  | Reverse | CAAGACCTTCAGCCGCCATC |
| DACH1 | Forward | CCATGAGCAACTATCATGCC |
|  | Reverse | TGTCCATGCCCAGTTAGAGA |
| SLCO3A1 | Forward | CATCGCGCTCAAATCCTTCG |
|  | Reverse | AATGTGATTCGGGGCAGGTT |
