# Supplementary material for: Comprehensive bioinformatics analysis of acquired progesterone resistance in endometrial cancer cell line
Source: J Transl Med. 2019 Feb 27;17:58. doi: 10.1186/s12967-019-1814-6 (PMC6391799; doi:10.1186/s12967-019-1814-6)
Supplement: Supplementary file 3 — Additional file 3: Table S3. Top 30 DEGs sorted by |log2foldchange|. [file 12967_2019_1814_MOESM3_ESM.docx]

Additional Table S3. Top 30 DEGs sorted by |log2foldchang|. FC: fold change.

| Gene symbol | Log2FC | P-value | Description |
| --- | --- | --- | --- |
| VIM | 11.8094267 | 6.6463E-07 | up-regulated |
| EFEMP1 | 11.2473377 | 1.0331E-08 | up-regulated |
| SPON1 | 10.9760748 | 7.4216E-09 | down-regulated |
| ANO1 | 10.7788283 | 2.0379E-09 | down-regulated |
| CPS1 | 10.61286033 | 4.5503E-09 | up-regulated |
| SPINK13 | 10.49912787 | 1.2162E-08 | up-regulated |
| CLDN3 | 10.47862787 | 7.527E-08 | down-regulated |
| SPRY1 | 10.44180063 | 1.56E-11 | down-regulated |
| NPTX1 | 10.41187473 | 9.38E-11 | up-regulated |
| MT1M | 10.367251 | 6.2679E-06 | up-regulated |
| TM4SF1 | 10.2739146 | 7.839E-10 | up-regulated |
| KCNE3 | 10.23742827 | 7.1088E-08 | down-regulated |
| FRG2C | 10.23559453 | 2.8989E-07 | down-regulated |
| LPL | 9.974120733 | 4.7985E-09 | up-regulated |
| WNT5A | 9.7983378 | 5.1935E-08 | up-regulated |
| HMGA2 | 9.736376633 | 4.096E-10 | down-regulated |
| NNMT | 9.705901233 | 1.063E-06 | up-regulated |
| LY6K | 9.695994367 | 7.3423E-08 | up-regulated |
| CSGALNACT1 | 9.691904333 | 5.0758E-08 | up-regulated |
| SHISA3 | 9.586970467 | 3.073E-09 | down-regulated |
| CDH13 | 9.576249233 | 2.7E-11 | up-regulated |
| SSTR1 | 9.569390733 | 1.0176E-09 | up-regulated |
| DUSP6 | 9.539429967 | 1.0086E-08 | down-regulated |
| NPAS3 | 9.4033933 | 8.3154E-09 | down-regulated |
| BCAT1 | 9.324751967 | 4.1E-11 | down-regulated |
| FAM179A | 9.278841733 | 2.8304E-08 | up-regulated |
| EMX2 | 9.2715345 | 3.1118E-05 | down-regulated |
| CARD16 | 9.262431333 | 6.3979E-07 | up-regulated |
| TM4SF1 | 9.196679567 | 4.3575E-08 | up-regulated |
| CT83 | 9.030759733 | 1.715E-10 | up-regulated |
