# Supplementary material for: Comprehensive bioinformatics analysis of acquired progesterone resistance in endometrial cancer cell line
Source: J Transl Med. 2019 Feb 27;17:58. doi: 10.1186/s12967-019-1814-6 (PMC6391799; doi:10.1186/s12967-019-1814-6)
Supplement: Supplementary file 4 — Additional file 4: Table S4. Microarray analysis result of genes related to lipid metabolic and biosynthetic process in IshikawaPR and Ishikawa cell. [file 12967_2019_1814_MOESM4_ESM.docx]

**Additional Table S4**. Microarray analysis result of genes related to lipid metabolic and biosynthetic process in IshikawaPR and Ishikawa cell.

| **Description** | **Gene name** | **Fold Change** | **P-value** |
| --- | --- | --- | --- |
| fatty acid biosynthetic process(GO:0006633) | EDN1 | 4.2392645 | 6.01857E-05 |
|  | EDN2 | 19.728059 | 3.33047E-08 |
|  | PTGS1 | 87.564636 | 6.3759E-08 |
|  | PTGES2 | 2.10211 | 0.002047816 |
|  | PTGES | 217.52912 | 2.0287E-09 |
|  | FADS1 | 3.02554 | 2.50723E-06 |
|  | FADS3 | 5.9524288 | 2.59554E-06 |
|  | ELOVL3 | 5.0537579 | 3.60583E-06 |
|  | ALOX5 | 18.409216 | 7.24401E-06 |
|  | PLA2G5 | 2.2281427 | 0.000194897 |
|  | RNPEP | 2.0833323 | 1.19861E-06 |
|  | GGTLC2 | 2.0793973 | 0.000346448 |
|  | GGTLC1 | 3.0114535 | 1.93148E-05 |
|  | AKR1C3 | 14.821332 | 4.45103E-07 |
|  | SIRT1 | 2.3285938 | 4.40425E-05 |
|  | ACADVL | 2.0164906 | 6.1286E-07 |
|  | BRCA1 | 2.6093962 | 9.05638E-06 |
|  | NR1H3 | 2.192331 | 3.13839E-06 |
|  | AGT | 2.0097983 | 2.80247E-05 |
|  | APOC2 | 2.1354492 | 0.000427835 |
|  | MID1IP1 | 2.2387943 | 1.99964E-06 |
|  | ALOX15 | 1.4664034 | 0.003796253 |
|  | MGLL | 46.754364 | 2.7798E-09 |
|  | PTPLAD2 | 2.3126797 | 0.000118877 |
|  | LPL | 1005.7951 | 4.7985E-09 |
|  | OLAH | 41.995314 | 3.39765E-06 |
|  | PRKAA1 | 2.371464 | 0.000919122 |
|  | FA2H | 78.932064 | 3.42627E-08 |
|  | PTPLA | 1.0578952 | 0.354797175 |
| lipid metabolic process(GO:0006629) | ACOT1 | 33.074839 | 1.93386E-07 |
|  | AKR1C4 | 16.353343 | 2.16604E-06 |
|  | ADH4 | 4.7334753 | 0.000499611 |
|  | AKR1C1 | 1.5618768 | 0.124411043 |
|  | GPC5 | 9.4601361 | 5.87913E-05 |
|  | HSPG2 | 1.1375207 | 0.019172185 |
|  | LRP1 | 3.758053 | 1.88266E-05 |
|  | LRP2 | 2.4068674 | 0.000175833 |
|  | AKR1B10 | 2.7962033 | 0.000541147 |
|  | RARRES2 | 3.3058093 | 0.000732439 |
|  | RBP4 | 58.195001 | 4.15716E-08 |
|  | RDH5 | 2.1758847 | 0.000444607 |
|  | STRA6 | 2.6208152 | 0.000169019 |
|  | LRP8 | 2.3502347 | 0.000627109 |
|  | DHRS3 | 2.7683672 | 0.000187165 |
|  | A4GALT | 2.1655673 | 7.01099E-05 |
|  | CYP4A11 | 5.569543 | 2.25213E-05 |
|  | PAM | 1.5008897 | 0.010645985 |
|  | ACSL5 | 2.3720894 | 3.87562E-05 |
|  | ALDH1A3 | 2.1858749 | 7.35051E-05 |
|  | DHDDS | 1.9035502 | 4.8361E-06 |
| lipid translocation(GO:0034204) | ATP8B3 | 18.789787 | 6.22808E-06 |
|  | ABCA1 | 1.4139126 | 0.291309062 |
|  | ATP11A | 6.5885085 | 6.42861E-07 |
|  | ATP9B | 2.6442263 | 5.59007E-07 |
|  | KCNN4 | 18.809271 | 5.57248E-05 |
|  | ATP10D | 5.9681398 | 3.94723E-05 |
| regulation of lipid metabolic process(GO:0019216) | IGFBP7 | 66.12023 | 1.7336E-07 |
|  | PDHA1 | 2.6103507 | 3.52609E-06 |
|  | PDHB | 2.379314 | 3.19608E-06 |
|  | PDP2 | 2.0915434 | 4.04875E-05 |
|  | PDHX | 2.1008604 | 0.000171869 |
|  | ZP3 | 2.047779 | 0.003278647 |
| lipid transport(GO:0006869) | IRS2 | 9.4784293 | 6.9093E-06 |
|  | ABCA7 | 4.1879051 | 8.86333E-06 |
| lipid biosynthetic process(GO:0008610) | PTGES3 | -2.156985 | 4.96896E-05 |
|  | FAM213B | -2.064363 | 9.9951E-06 |
|  | ST8SIA2 | -2.898882 | 0.000332986 |
|  | RDH10 | -4.654118 | 7.66314E-07 |
|  | PGAP2 | -2.00467 | 6.53511E-05 |
|  | PIGF | -2.740024 | 5.83918E-06 |
|  | PIGH | -2.780733 | 4.38919E-05 |
|  | PIGY | -3.638277 | 1.81041E-05 |
|  | PIGL | -4.89528 | 1.42381E-05 |
|  | FAXDC2 | -2.232555 | 0.000304975 |
|  | FASN | -2.365857 | 3.62092E-05 |
|  | ACACB | -5.512722 | 2.14524E-05 |
|  | AGMO | -2.350779 | 0.000443487 |
|  | LIPC | -3.911395 | 0.000150278 |
|  | HSD17B12 | -1.416338 | 4.82103E-05 |
|  | PRKAG2 | -4.07004 | 1.60085E-06 |
|  | PRKAA2 | -2.459568 | 0.000695663 |
|  | MSMO1 | -6.651219 | 2.11589E-06 |
|  | SC5D | -3.620629 | 4.43727E-05 |
|  | ELOVL4 | -33.11116 | 8.64475E-06 |
|  | CH25H | -3.754299 | 0.00565472 |
|  | ELOVL2 | -6.305497 | 0.000336648 |
|  | FADS2 | -2.674647 | 2.2297E-05 |
|  | CHKB | -3.359595 | 2.58005E-05 |
|  | SLC27A1 | -7.487875 | 1.33822E-06 |
|  | EPT1 | -2.75026 | 1.46228E-05 |
|  | MOGAT1 | -10.70689 | 1.4552E-05 |
|  | GPD1L | -3.206799 | 3.0635E-06 |
|  | PLA2G4D | -4.159327 | 0.002596131 |
|  | AGPAT5 | -2.346223 | 1.84978E-06 |
|  | AGPAT3 | -2.479228 | 1.36374E-05 |
|  | PLA2G12A | -2.928023 | 3.53467E-07 |
|  | PLA2G10 | -2.125028 | 0.008033268 |
|  | PEMT | -3.142512 | 2.55992E-05 |
|  | CHAT | -2.353027 | 0.00101845 |
|  | FABP5 | -4.228241 | 8.44414E-06 |
|  | PCYT1A | -3.610087 | 1.84909E-05 |
|  | SLC44A2 | -2.299891 | 0.00485153 |
|  | CDS1 | -19.96155 | 6.63006E-05 |
|  | PTDSS1 | -2.621176 | 3.10358E-06 |
|  | SH3YL1 | -17.65592 | 3.77636E-07 |
|  | PIK3CG | -2.185926 | 0.000370964 |
|  | PIK3R1 | -9.174559 | 1.13055E-05 |
|  | PLCG2 | -15.14086 | 2.04956E-05 |
|  | PIK3R3 | -8.247881 | 3.86677E-07 |
|  | INPP4B | -1.733526 | 0.00600538 |
|  | MTMR7 | -10.87907 | 2.59434E-06 |
|  | TPTE2 | -2.173128 | 0.000275566 |
|  | CD81 | -2.54681 | 4.54632E-05 |
|  | FIG4 | -2.200203 | 1.8167E-05 |
|  | LPCAT2 | -3.117817 | 0.012173573 |
|  | SPHK2 | -1.905571 | 0.067801743 |
|  | UGT8 | -5.303102 | 8.4492E-07 |
|  | SPTLC1 | -1.272535 | 0.010539294 |
|  | ST8SIA5 | -2.182877 | 0.002275607 |
|  | ST8SIA1 | -5.757505 | 1.80287E-05 |
|  | HSD17B11 | -11.02573 | 9.03241E-07 |
|  | PRLR | -6.785061 | 3.76302E-05 |
|  | HINT2 | -2.229385 | 1.00843E-05 |
|  | DHCR7 | -4.124928 | 3.48015E-06 |
|  | HMGCR | -4.879572 | 8.02608E-07 |
|  | LSS | -2.239507 | 1.55419E-05 |
|  | MVK | -2.266072 | 0.00018694 |
|  | HSD17B7 | -1.49638 | 0.000451853 |
|  | SQLE | -3.833935 | 4.28225E-05 |
|  | TM7SF2 | -2.257472 | 8.25411E-05 |
|  | IDI2 | -2.104107 | 0.000291713 |
|  | SLC27A5 | -2.003588 | 5.19456E-06 |
|  | CYP27A1 | -16.44036 | 9.21952E-06 |
|  | ACOX2 | -13.34018 | 5.02709E-06 |
|  | HSD17B6 | -2.573819 | 2.60493E-05 |
|  | HSD11B1 | -2.009126 | 0.006060171 |
|  | PDSS1 | -2.517742 | 0.000285954 |
|  | PEX7 | -2.75548 | 1.14174E-07 |
|  | FAR1 | -1.103081 | 0.403004026 |
|  | ISYNA1 | -6.262553 | 2.96879E-07 |
|  | SERAC1 | -2.322522 | 0.000386193 |
|  | CMAS | -4.289752 | 4.64147E-06 |
|  | B3GNT5 | -2.163395 | 1.37925E-05 |
|  | LIPT2 | -2.574728 | 1.26951E-06 |
|  | HTR2B | -2.249167 | 0.024045475 |
|  | HTR2C | -10.25869 | 1.29705E-05 |
|  | LDLR | -2.637407 | 1.6413E-07 |
|  | PLIN5 | -3.124214 | 6.84E-05 |
|  | GFI1 | -3.725865 | 5.82586E-08 |
|  | SNAI1 | -2.943875 | 0.000790264 |
|  | APOE | -8.524593 | 5.75769E-07 |
|  | C14ORF1 | -2.859068 | 7.87432E-06 |
|  | CYB5R2 | -97.94266 | 1.67206E-05 |
|  | CYB5R1 | -2.42144 | 4.25976E-07 |
|  | PIGP | -2.386948 | 8.83393E-05 |
|  | PIGK | -2.128151 | 5.37456E-06 |
|  | ELOVL1 | -2.174393 | 0.000116506 |
|  | ELOVL6 | -11.10071 | 1.04424E-07 |
|  | ELOVL7 | -2.954879 | 1.31012E-05 |
|  | GGT1 | -2.019966 | 0.000221314 |
|  | GGT3P | -2.072488 | 0.00053885 |
|  | MGST2 | -2.082492 | 9.23999E-06 |
|  | RNPEPL1 | -2.730827 | 2.13574E-05 |
|  | SYK | -19.38581 | 3.73655E-06 |
|  | ACSL1 | -2.400709 | 2.31548E-05 |
|  | ACSL4 | -3.512928 | 5.48412E-05 |
|  | ACLY | -2.234873 | 4.85483E-05 |
|  | ACSS1 | -4.437498 | 5.91509E-05 |
|  | COL4A3BP | -7.103331 | 1.47293E-08 |
|  | CSNK1G2 | -2.936546 | 0.00014077 |
|  | PPM1L | -2.261989 | 0.008916957 |
|  | CERS6 | -6.623383 | 1.23321E-05 |
|  | ACER2 | -1.484762 | 0.000525169 |
|  | CERS4 | -4.283826 | 8.05254E-05 |
|  | PPAP2C | -2.797719 | 0.000125718 |
|  | PPAP2B | -7.353208 | 6.89381E-06 |
|  | VAPA | -1.70818 | 4.08742E-07 |
|  | MAPK9 | -1.517353 | 0.003434748 |
|  | CRLS1 | -3.16548 | 4.7104E-08 |
|  | PLA2G6 | -3.738871 | 0.011706606 |
|  | DKK3 | -2.895331 | 6.30727E-05 |
|  | CYP2R1 | -35.93201 | 7.83668E-07 |
|  | CYP3A4 | -3.029572 | 0.000929429 |
|  | STK11 | -5.87119 | 4.36872E-07 |
|  | CYP1A1 | -3.112019 | 0.000108984 |
|  | SOD1 | -2.018982 | 0.000188626 |
|  | APOC1 | -5.657028 | 3.49896E-06 |
|  | PLA2G4E | -2.931498 | 3.91927E-05 |
|  | PLA2G3 | -8.921537 | 0.000674559 |
|  | GPCPD1 | -10.04168 | 2.2245E-06 |
|  | MBOAT7 | -2.166508 | 0.000142617 |
|  | PLBD1 | -2.976373 | 8.37643E-06 |
|  | SMPD2 | -2.72515 | 1.0526E-05 |
|  | FGF19 | -18.16901 | 0.000456289 |
