# Supplementary material for: Comprehensive bioinformatics analysis of acquired progesterone resistance in endometrial cancer cell line
Source: J Transl Med. 2019 Feb 27;17:58. doi: 10.1186/s12967-019-1814-6 (PMC6391799; doi:10.1186/s12967-019-1814-6)
Supplement: Supplementary file 5 — Additional file 5: Table S5. PGR co-expression genes within endometrial cancer were identified form cBioPortal. 859 significantly PGR correlated genes were selected. [file 12967_2019_1814_MOESM5_ESM.docx]

**Additional Table S5**. PGR co-expression genes within endometrial cancer were identified form cBioPortal. 859 significantly PGR correlated genes were selected with |Pearson score|>0.3 and |Spearman score|>0.3 as criterion.

| **Gene Symbol** | **Cytoband** | **Pearson Score** | **Spearman Score** |
| --- | --- | --- | --- |
| ABCF1 | 6p21.33 | -0.31 | -0.4 |
| ACADSB | 10q26.13 | 0.31 | 0.36 |
| ADA | 20q13.12 | -0.32 | -0.49 |
| ADD3 | 10q25.1-q25.2 | 0.35 | 0.39 |
| ADH5 | 4q23 | 0.38 | 0.33 |
| ALCAM | 3q13.11 | 0.35 | 0.44 |
| ABCD1 | Xq28 | -0.31 | -0.41 |
| ALDH3A2 | 17p11.2 | 0.39 | 0.47 |
| AKR1B1 | 7q33 | -0.33 | -0.47 |
| AMD1 | 6q21 | 0.31 | 0.34 |
| AMPD2 | 1p13.3 | -0.33 | -0.39 |
| AMPD3 | 11p15.4 | 0.32 | 0.37 |
| ANK3 | 10q21.2 | 0.5 | 0.53 |
| AR | Xq12 | 0.41 | 0.52 |
| ARL4D | 17q21.31 | 0.41 | 0.46 |
| MGC15885 | 15q22.2 | 0.34 | 0.4 |
| ARL1 | 12q23.2 | 0.36 | 0.43 |
| ART3 | 4q21.1\|4p15.1-p14 | 0.32 | 0.41 |
| ZFHX3 | 16q22.2-q22.3 | 0.4 | 0.47 |
| KIF1A | 2q37.3 | -0.31 | -0.36 |
| BAK1 | 6p21.31 | -0.36 | -0.47 |
| BBS2 | 16q13 | 0.33 | 0.42 |
| BBS4 | 15q24.1 | 0.32 | 0.42 |
| CEACAM1 | 19q13.2 | 0.33 | 0.51 |
| BMPR1B | 4q22.3 | 0.41 | 0.67 |
| BYSL | 6p21.1 | -0.34 | -0.46 |
| CAD | 2p23.3 | -0.32 | -0.45 |
| LIMS3-LOC440895 | 2q13 | 0.33 | 0.4 |
| ZBTB11-AS1 | 3q12.3 | -0.31 | -0.35 |
| CAPN6 | Xq23 | 0.37 | 0.37 |
| CCNG1 | 5q34 | 0.37 | 0.33 |
| ENTPD3 | 3p22.1 | 0.38 | 0.53 |
| LRBA | 4q31.3 | 0.31 | 0.41 |
| CDC20 | 1p34.2 | -0.32 | -0.42 |
| CDC25B | 20p13 | -0.42 | -0.61 |
| CDKN2A | 9p21.3 | -0.34 | -0.36 |
| CDS1 | 4q21.23 | 0.38 | 0.4 |
| CEBPB | 20q13.13 | -0.33 | -0.39 |
| TBCB | 19q13.12 | -0.32 | -0.4 |
| COL12A1 | 6q13-q14.1 | 0.45 | 0.46 |
| CRY2 | 11p11.2 | 0.36 | 0.45 |
| CSTF1 | 20q13.2-q13.31 | -0.35 | -0.46 |
| CYP2J2 | 1p32.1 | 0.42 | 0.31 |
| SPATA18 | 4q12 | 0.44 | 0.64 |
| DACH1 | 13q21.33 | 0.33 | 0.39 |
| DAXX | 6p21.32 | -0.38 | -0.49 |
| DBT | 1p21.2 | 0.32 | 0.31 |
| DDX6 | 11q23.3 | 0.33 | 0.35 |
| DMXL1 | 5q23.1 | 0.37 | 0.37 |
| DIO2 | 14q31.1 | 0.33 | 0.45 |
| DNAH9 | 17p12 | 0.33 | 0.45 |
| TRDMT1 | 10p13 | 0.55 | 0.52 |
| CPEB2 | 4p15.32 | 0.33 | 0.37 |
| EEF1A1 | 6q13 | 0.38 | 0.32 |
| EFNB1 | Xq13.1 | -0.31 | -0.33 |
| ELF1 | 13q14.11 | 0.38 | 0.39 |
| ELF2 | 4q31.1 | 0.31 | 0.35 |
| EPB41L2 | 6q23.1-q23.2 | 0.51 | 0.5 |
| EPHB2 | 1p36.12 | -0.39 | -0.52 |
| ERG | 21q22.2 | 0.34 | 0.41 |
| ESR1 | 6q25.1-q25.2 | 0.7 | 0.8 |
| EYA2 | 20q13.12 | 0.46 | 0.65 |
| FANCF | 11p14.3 | 0.34 | 0.39 |
| FBLN1 | 22q13.31 | 0.35 | 0.39 |
| FECH | 18q21.31 | 0.36 | 0.37 |
| FOXO3 | 6q21 | 0.4 | 0.37 |
| FOXO3B | 17p11.2 | 0.41 | 0.42 |
| FOLH1 | 11p11.12 | 0.32 | 0.5 |
| PRRC1 | 5q23.2 | 0.36 | 0.4 |
| GALK2 | 15q21.1-q21.2 | 0.42 | 0.4 |
| CAPSL | 5p13.2 | 0.32 | 0.51 |
| NIPSNAP2 | 7p11.2 | 0.32 | 0.35 |
| GCNT1 | 9q21.13 | 0.45 | 0.54 |
| GCNT2 | 6p24.3-p24.2 | 0.42 | 0.53 |
| GGTA1P | 9q33.2 | 0.41 | 0.51 |
| B4GALT1 | 9p21.1 | 0.45 | 0.49 |
| GCLC | 6p12.1 | 0.35 | 0.32 |
| GOLGB1 | 3q13.33 | 0.39 | 0.38 |
| ZNF474 | 5q23.2 | 0.34 | 0.49 |
| GRK6 | 5q35.3 | -0.31 | -0.36 |
| HADH | 4q25 | 0.43 | 0.48 |
| CDC20B | 5q11.2 | 0.32 | 0.56 |
| HMGA1 | 6p21.31 | -0.37 | -0.48 |
| FOXA2 | 20p11.21 | 0.38 | 0.57 |
| HOXA3 | 7p15.2 | -0.31 | -0.36 |
| HSD17B4 | 5q23.1 | 0.35 | 0.47 |
| PAPD4 | 5q14.1 | 0.36 | 0.33 |
| IGF1 | 12q23.2 | 0.43 | 0.5 |
| IHH | 2q35 | 0.49 | 0.72 |
| IL1R1 | 2q11.2-q12.1 | 0.31 | 0.33 |
| IL5RA | 3p26.2 | 0.4 | 0.49 |
| ITGA5 | 12q13.13 | -0.31 | -0.37 |
| STT3A | 11q24.2 | 0.33 | 0.42 |
| JUP | 17q21.2 | -0.33 | -0.34 |
| KCNE1 | 21q22.12 | 0.33 | 0.4 |
| KIFC1 | 6p21.32 | -0.32 | -0.36 |
| KPNA3 | 13q14.2 | 0.32 | 0.36 |
| KPNA5 | 6q22.1 | 0.37 | 0.37 |
| KRT7 | 12q13.13 | -0.31 | -0.39 |
| AFF3 | 2q11.2 | 0.41 | 0.5 |
| LAMP2 | Xq24 | 0.35 | 0.34 |
| LIMK1 | 7q11.23 | -0.37 | -0.49 |
| LIMS1 | 2q12.3 | 0.51 | 0.4 |
| HINT3 | 6q22.32 | 0.42 | 0.45 |
| SMAD9 | 13q13.3 | 0.5 | 0.37 |
| MATN1 | 1p35.2 | 0.49 | 0.4 |
| DACT2 | 6q27 | 0.45 | 0.45 |
| MCM2 | 3q21.3 | -0.34 | -0.38 |
| MCM5 | 22q12.3 | -0.33 | -0.39 |
| MCM7 | 7q22.1 | -0.31 | -0.37 |
| DNAJB9 | 7q31.1\|14q24.2-q24.3 | 0.34 | 0.35 |
| MDM2 | 12q15 | 0.4 | 0.47 |
| C6ORF118 | 6q27 | 0.35 | 0.46 |
| MIA2 | 14q21.1 | 0.57 | 0.57 |
| AFDN | 6q27 | 0.42 | 0.42 |
| ALDH6A1 | 14q24.3 | 0.51 | 0.46 |
| MUC7 | 4q13.3 | 0.38 | 0.44 |
| MYB | 6q23.3 | 0.35 | 0.43 |
| NAB2 | 12q13.3 | -0.34 | -0.45 |
| NEK1 | 4q33 | 0.49 | 0.46 |
| NFIC | 19p13.3 | 0.35 | 0.34 |
| NFIX | 19p13.13 | 0.31 | 0.35 |
| NHS | Xp22.2-p22.13 | 0.33 | 0.42 |
| NMT1 | 17q21.31 | -0.31 | -0.36 |
| SLC11A2 | 12q13.12 | 0.45 | 0.53 |
| NT5E | 6q14.3 | 0.42 | 0.37 |
| ROR1 | 1p31.3 | 0.42 | 0.31 |
| NUCB2 | 11p15.1 | 0.38 | 0.5 |
| ODF2 | 9q34.11 | 0.34 | 0.39 |
| KLLN | 10q23.31 | 0.32 | 0.38 |
| P2RY1 | 3q25.2 | 0.32 | 0.33 |
| PAM | 5q21.1 | 0.38 | 0.49 |
| PCM1 | 8p22 | 0.4 | 0.41 |
| ENPP3 | 6q23.2 | 0.35 | 0.52 |
| PEX1 | 7q21.2 | 0.34 | 0.32 |
| PEX7 | 6q23.3 | 0.4 | 0.37 |
| PFDN4 | 20q13.2 | -0.32 | -0.53 |
| C7ORF57 | 7p12.3 | 0.35 | 0.4 |
| PGM3 | 6q14.1 | 0.31 | 0.35 |
| PHKB | 16q12.1 | 0.31 | 0.48 |
| PIGH | 14q24.1 | 0.32 | 0.33 |
| SMIM14 | 4p14 | 0.31 | 0.42 |
| PIK3R1 | 5q13.1 | 0.33 | 0.61 |
| PIP4K2A | 10p12.2 | 0.37 | 0.55 |
| PLCB4 | 20p12.3-p12.2 | 0.46 | 0.54 |
| UBL3 | 13q12.3 | 0.35 | 0.46 |
| PODXL | 7q32.3 | 0.34 | 0.37 |
| PPM1G | 2p23.3 | -0.35 | -0.43 |
| PPP2R1B | 11q23.1 | 0.35 | 0.46 |
| PPP2R2C | 4p16.1 | 0.41 | 0.34 |
| PRKCH | 14q23.1 | 0.36 | 0.43 |
| MAPK6 | 15q21.2 | 0.31 | 0.38 |
| DNAJC3 | 13q32.1 | 0.38 | 0.51 |
| PRLR | 5p13.2 | 0.39 | 0.59 |
| CCDC125 | 5q13.2 | 0.45 | 0.58 |
| PSMC5 | 17q23.3 | -0.31 | -0.32 |
| PSMD2 | 3q27.1 | -0.36 | -0.41 |
| TWF1 | 12q12 | 0.39 | 0.44 |
| PEX2 | 8q21.13 | 0.46 | 0.39 |
| PZP | 12p13.31 | 0.36 | 0.56 |
| RAD51B | 14q24.1 | 0.37 | 0.34 |
| TCTE1 | 6p21.1 | 0.34 | 0.52 |
| RANBP1 | 22q11.21 | -0.32 | -0.38 |
| RANBP2 | 2q13 | 0.49 | 0.34 |
| RANGAP1 | 22q13.2 | -0.31 | -0.46 |
| RARA | 17q21.2 | 0.34 | 0.42 |
| RARG | 12q13.13 | -0.36 | -0.44 |
| RBBP7 | Xp22.2 | 0.37 | 0.52 |
| RBL2 | 16q12.2 | 0.35 | 0.37 |
| GLIS3 | 9p24.2 | 0.34 | 0.44 |
| RELB | 19q13.32 | -0.31 | -0.37 |
| REV3L | 6q21 | 0.36 | 0.4 |
| RFC2 | 7q11.23 | -0.33 | -0.39 |
| RFC4 | 3q27.3 | -0.32 | -0.38 |
| RFX3 | 9p24.2 | 0.41 | 0.39 |
| RNF6 | 13q12.13 | 0.32 | 0.41 |
| RP1 | 8q11.23-q12.1 | 0.34 | 0.4 |
| RREB1 | 6p24.3 | 0.31 | 0.33 |
| SLC25A35 | 17p13.1 | 0.32 | 0.47 |
| ATXN1 | 6p22.3 | 0.32 | 0.36 |
| ATXN7 | 3p14.1 | 0.35 | 0.33 |
| SEC14L1 | 17q25.2-q25.3 | 0.38 | 0.45 |
| SGCB | 4q12 | 0.36 | 0.54 |
| SGCD | 5q33.2-q33.3 | 0.32 | 0.52 |
| SH3BGR | 21q22.2 | 0.38 | 0.49 |
| SH3BGRL | Xq21.1 | 0.33 | 0.34 |
| ZNF321P | 19q13.41 | 0.31 | 0.48 |
| TUBB | 6p21.33 | -0.31 | -0.37 |
| SLC22A5 | 5q31.1 | 0.58 | 0.59 |
| C9ORF72 | 9p21.2 | 0.44 | 0.46 |
| CCDC171 | 9p22.3 | 0.31 | 0.41 |
| SOX5 | 12p12.1 | 0.37 | 0.45 |
| UAP1 | 1q23.3 | 0.41 | 0.34 |
| AURKA | 20q13.2 | -0.38 | -0.47 |
| SYPL1 | 7q22.3 | 0.31 | 0.32 |
| TCOF1 | 5q32-q33.1 | -0.32 | -0.42 |
| TEAD4 | 12p13.33 | -0.32 | -0.44 |
| TEC | 4p12-p11 | 0.36 | 0.49 |
| TEK | 9p21.2 | 0.34 | 0.38 |
| TFDP2 | 3q23 | -0.31 | -0.36 |
| TK1 | 17q25.3 | -0.31 | -0.37 |
| TMF1 | 3p14.1 | 0.34 | 0.36 |
| TPD52L2 | 20q13.33 | -0.35 | -0.45 |
| ADAMTS19 | 5q23.3 | 0.41 | 0.6 |
| TRAF5 | 1q32.3 | 0.35 | 0.35 |
| TRPS1 | 8q23.3 | 0.42 | 0.4 |
| TTC4 | 1p32.3 | -0.34 | -0.4 |
| TXK | 4p12 | 0.37 | 0.5 |
| UPK1B | 3q13.32 | 0.34 | 0.46 |
| UTRN | 6q24.2 | 0.31 | 0.32 |
| VARS | 6p21.33 | -0.34 | -0.44 |
| VIM | 10p13 | 0.45 | 0.4 |
| CLIP2 | 7q11.23 | -0.35 | -0.5 |
| VMAC | 19p13.3 | 0.34 | 0.61 |
| WEE1 | 11p15.4 | 0.32 | 0.32 |
| WRB | 21q22.2 | 0.34 | 0.35 |
| XBP1 | 22q12.1\|22q12 | 0.38 | 0.48 |
| XIST | Xq13.2 | 0.34 | 0.33 |
| ZNF708 | 19p12 | 0.33 | 0.35 |
| ZNF33B | 10q11.21 | 0.34 | 0.41 |
| ZNF204P | 6p22.1 | 0.39 | 0.4 |
| ZNF224 | 19q13.31 | 0.32 | 0.31 |
| PAX8 | 2q14.1 | -0.31 | -0.35 |
| BAG6 | 6p21.33 | -0.33 | -0.39 |
| C5ORF63 | 5q23.2 | 0.33 | 0.35 |
| SHOC2 | 10q25.2 | 0.39 | 0.45 |
| CUL5 | 11q22.3 | 0.31 | 0.33 |
| PTP4A2 | 1p35.2 | -0.32 | -0.32 |
| MLF2 | 12p13.31 | -0.33 | -0.38 |
| IFT88 | 13q12.11 | 0.36 | 0.41 |
| NRIP1 | 21q11.2-q21.1 | 0.54 | 0.52 |
| HIPK1 | 1p13.2 | 0.42 | 0.53 |
| ACOX3 | 4p16.1 | 0.38 | 0.38 |
| CDC45 | 22q11.21 | -0.33 | -0.41 |
| FZD6 | 8q22.3 | 0.38 | 0.43 |
| C9ORF152 | 9q31.3 | 0.36 | 0.63 |
| NME5 | 5q31.2 | 0.36 | 0.53 |
| RASAL1 | 12q24.13 | -0.32 | -0.44 |
| SORBS2 | 4q35.1 | 0.5 | 0.66 |
| RAE1 | 20q13.31 | -0.34 | -0.47 |
| SLC43A1 | 11q12.1 | 0.56 | 0.49 |
| DGKE | 17q22 | 0.38 | 0.49 |
| AGPS | 2q31.2 | 0.47 | 0.35 |
| CDC14A | 1p21.2 | 0.64 | 0.63 |
| PDXK | 21q22.3 | -0.34 | -0.39 |
| PLPP2 | 19p13.3 | 0.36 | 0.56 |
| USO1 | 4q21.1 | 0.39 | 0.36 |
| LAMTOR3 | 4q23 | 0.36 | 0.35 |
| NUMB | 14q24.2-q24.3 | 0.41 | 0.42 |
| GALNT4 | 12q21.33 | 0.68 | 0.73 |
| CD164 | 6q21 | 0.34 | 0.32 |
| SNAP23 | 15q15.1-q15.2 | 0.33 | 0.37 |
| FPGT | 1p31.1 | 0.36 | 0.43 |
| SUCLG2 | 3p14.1 | 0.32 | 0.36 |
| DCAF5 | 14q24.1 | 0.35 | 0.4 |
| KAT2B | 3p24.3 | 0.42 | 0.43 |
| UBA3 | 3p14.1 | 0.41 | 0.41 |
| PAPSS1 | 4q25 | 0.38 | 0.52 |
| LDB2 | 4p15.32 | 0.34 | 0.37 |
| USP8 | 15q21.2 | 0.33 | 0.35 |
| MTMR6 | 13q12.13 | 0.32 | 0.36 |
| USF3 | 3q13.2 | 0.46 | 0.41 |
| REPS2 | Xp22.2 | 0.38 | 0.58 |
| BMP15 | Xp11.22 | 0.35 | 0.35 |
| AURKB | 17p13.1 | -0.34 | -0.42 |
| LARGE1 | 22q12.3 | 0.31 | 0.46 |
| VAPB | 20q13.32 | -0.34 | -0.41 |
| MAGI1 | 3p14.1 | 0.54 | 0.5 |
| RPS6KA5 | 14q32.11 | 0.48 | 0.45 |
| TCEAL1 | Xq22.2 | 0.45 | 0.37 |
| UBE4A | 11q23.3 | 0.34 | 0.33 |
| RAB28 | 4p15.33 | 0.48 | 0.48 |
| SLC9A3R1 | 17q25.1 | 0.34 | 0.35 |
| ASB6 | 9q34.11 | -0.31 | -0.38 |
| FAM189A2 | 9q21.12 | 0.49 | 0.59 |
| ZNF264 | 19q13.43 | 0.34 | 0.37 |
| NTN1 | 17p13.1 | 0.46 | 0.4 |
| MED23 | 6q23.2 | 0.4 | 0.35 |
| HOMER2 | 15q25.2 | 0.42 | 0.57 |
| CHST3 | 10q22.1 | 0.39 | 0.33 |
| THEMIS2 | 1p35.3 | -0.31 | -0.43 |
| PIGB | 15q21.3 | 0.38 | 0.44 |
| SCAMP1 | 5q14.1 | 0.46 | 0.43 |
| MTRF1 | 13q14.11 | 0.32 | 0.37 |
| GNA14 | 9q21.2 | 0.38 | 0.47 |
| SOGA1 | 20q11.23 | -0.34 | -0.37 |
| ZNF516 | 18q23 | 0.55 | 0.61 |
| DZIP3 | 3q13.13 | 0.33 | 0.38 |
| GREB1 | 2p25.1 | 0.68 | 0.65 |
| RAPGEF2 | 4q32.1 | 0.34 | 0.31 |
| ESPL1 | 12q13.13 | -0.32 | -0.4 |
| HERPUD1 | 16q13 | 0.43 | 0.53 |
| SEMA3E | 7q21.11 | 0.4 | 0.55 |
| CCP110 | 16p12.3 | 0.32 | 0.36 |
| ARHGAP32 | 11q24.3 | 0.42 | 0.46 |
| LINC00261 | 20p11.21 | 0.38 | 0.62 |
| ZFYVE16 | 5q14.1 | 0.33 | 0.32 |
| KIAA0232 | 4p16.1 | 0.33 | 0.41 |
| DELE1 | 5q31.3 | 0.33 | 0.43 |
| MFAP3L | 4q33 | 0.34 | 0.5 |
| ZNF518A | 10q24.1 | 0.33 | 0.34 |
| PJA2 | 5q21.3 | 0.32 | 0.37 |
| SEC24D | 4q26 | 0.32 | 0.36 |
| TBC1D4 | 13q22.2 | 0.32 | 0.33 |
| RHOBTB1 | 10q21.2 | 0.5 | 0.44 |
| HS3ST1 | 4p15.33 | 0.41 | 0.45 |
| PDCD6IP | 3p22.3 | 0.31 | 0.36 |
| TOM1L1 | 17q22 | 0.36 | 0.36 |
| ABCF2 | 7q36.1 | -0.32 | -0.42 |
| APOBEC4 | 1q25.3 | 0.41 | 0.53 |
| RBM7 | 11q23.2 | 0.47 | 0.42 |
| RBM5 | 3p21.31 | 0.32 | 0.39 |
| ALG3 | 3q27.1 | -0.31 | -0.42 |
| MPZL2 | 11q23.3 | 0.31 | 0.53 |
| TRIM13 | 13q14.2 | 0.34 | 0.38 |
| STX6 | 1q25.3 | 0.32 | 0.42 |
| SPRY3 | Xq28 and Yq12 | 0.43 | 0.55 |
| STAM2 | 2q23.3 | 0.4 | 0.39 |
| DENND4A | 15q22.31 | 0.41 | 0.37 |
| BET1 | 7q21.3 | 0.45 | 0.35 |
| RGS19 | 20q13.33 | -0.32 | -0.41 |
| DNAJA2 | 16q11.2 | 0.38 | 0.38 |
| ATP8A1 | 4p13 | 0.45 | 0.41 |
| PIAS3 | 1q21.1 | 0.43 | 0.55 |
| N4BP2L2 | 13q13.1 | 0.33 | 0.4 |
| ACAA2 | 18q21.1 | -0.34 | -0.43 |
| SLC30A9 | 4p13 | 0.47 | 0.46 |
| ZBTB18 | 1q44 | 0.44 | 0.48 |
| NXF1 | 11q12.3 | 0.31 | 0.32 |
| ARL6IP5 | 3p14.1 | 0.54 | 0.53 |
| PDLIM5 | 4q22.3 | 0.34 | 0.44 |
| TRIM3 | 11p15.4 | 0.33 | 0.36 |
| IGF2BP2 | 3q27.2 | -0.34 | -0.49 |
| PRELID3A | 18p11.21 | -0.31 | -0.41 |
| GNB5 | 15q21.2 | 0.47 | 0.58 |
| MGEA5 | 10q24.32 | 0.31 | 0.31 |
| GIPC1 | 19p13.12 | -0.35 | -0.37 |
| TRAF3IP2 | 6q21 | 0.55 | 0.64 |
| AHCYL1 | 1p13.3 | 0.4 | 0.48 |
| IQGAP2 | 5q13.3 | 0.36 | 0.47 |
| PGRMC1 | Xq24 | 0.36 | 0.42 |
| TMED2 | 12q24.31 | 0.4 | 0.39 |
| TMED10 | 14q24.3 | 0.4 | 0.49 |
| KRTAP5-AS1 | 11p15.5 | 0.32 | 0.43 |
| ILVBL | 19p13.12 | -0.31 | -0.44 |
| KIF2C | 1p34.1 | -0.32 | -0.39 |
| SLC35D2 | 9q22.32 | 0.4 | 0.33 |
| ADRM1 | 20q13.33 | -0.32 | -0.55 |
| OGFR | 20q13.33 | -0.31 | -0.39 |
| UBE2C | 20q13.12 | -0.32 | -0.44 |
| KLK12 | 19q13.41 | 0.38 | 0.41 |
| ADAMTS8 | 11q24.3 | 0.42 | 0.42 |
| C12ORF74 | 12q22 | 0.34 | 0.54 |
| ADAMTS6 | 5q12.3 | 0.49 | 0.58 |
| BAZ1A | 14q13.1-q13.2 | 0.41 | 0.32 |
| POLI | 18q21.2 | 0.38 | 0.45 |
| MGAT4A | 2q11.2 | -0.31 | -0.36 |
| TMC6 | 17q25.3 | -0.37 | -0.39 |
| ACOT7 | 1p36.31 | -0.35 | -0.54 |
| FAM92B | 16q24.1 | 0.31 | 0.41 |
| FRMPD2 | 10q11.22 | 0.36 | 0.47 |
| DYDC1 | 10q23.1 | 0.35 | 0.35 |
| KBTBD3 | 11q22.3 | 0.32 | 0.37 |
| SPINDOC | 11q13.1 | -0.31 | -0.37 |
| WDR66 | 12q24.31 | 0.31 | 0.35 |
| DCDC1 | 11p13 | 0.36 | 0.38 |
| OTUB2 | 14q32.12 | 0.33 | 0.38 |
| C17ORF53 | 17q21.31 | -0.33 | -0.38 |
| MLPH | 2q37.3 | 0.37 | 0.57 |
| CENPO | 2p23.3 | -0.37 | -0.4 |
| FAM216B | 13q14.11 | 0.44 | 0.53 |
| TMEM231 | 16q23.1 | 0.33 | 0.45 |
| ARMT1 | 6q25.1 | 0.37 | 0.34 |
| CCDC121 | 2p23.3 | 0.36 | 0.31 |
| B3GLCT | 13q12.3 | 0.38 | 0.45 |
| MCPH1 | 8p23.1 | 0.31 | 0.36 |
| RHBDF2 | 17q25.1 | -0.31 | -0.37 |
| NEIL1 | 15q24.2 | 0.33 | 0.41 |
| PLEKHG7 | 12q22 | 0.33 | 0.62 |
| ICE2 | 15q22.2 | 0.37 | 0.41 |
| KLF3-AS1 | 4p14 | 0.31 | 0.4 |
| TEX9 | 15q21.3 | 0.34 | 0.4 |
| DTD2 | 14q12 | 0.36 | 0.37 |
| NSUN7 | 4p14 | 0.32 | 0.32 |
| TTLL7 | 1p31.1 | 0.34 | 0.46 |
| CCDC88C | 14q32.11-q32.12 | 0.42 | 0.44 |
| WDR78 | 1p31.3 | 0.35 | 0.48 |
| MRAP2 | 6q14.2 | 0.5 | 0.57 |
| JHY | 11q24.1 | 0.36 | 0.4 |
| ZC3H14 | 14q31.3 | 0.49 | 0.38 |
| PYROXD1 | 12p12.1 | 0.33 | 0.35 |
| SPEF2 | 5p13.2 | 0.36 | 0.52 |
| TUBA3E | 2q21.1 | 0.34 | 0.64 |
| PLPPR3 | 19p13.3 | 0.31 | 0.34 |
| NOL10 | 2p25.1 | -0.34 | -0.44 |
| GRHL2 | 8q22.3 | 0.37 | 0.51 |
| DSN1 | 20q11.23 | -0.31 | -0.4 |
| ZNF702P | 19q13.41 | 0.39 | 0.36 |
| TRAPPC13 | 5q12.3 | 0.33 | 0.36 |
| DGLUCY | 14q32.11 | 0.32 | 0.32 |
| FBXL18 | 7p22.1 | -0.33 | -0.42 |
| MAP3K19 | 2q21.3 | 0.37 | 0.54 |
| BBOF1 | 14q24.3 | 0.38 | 0.5 |
| CCDC170 | 6q25.1 | 0.52 | 0.68 |
| MROH9 | 1q24.3 | 0.47 | 0.53 |
| ZNF703 | 8p11.23 | 0.33 | 0.36 |
| ASRGL1 | 11q12.3 | 0.49 | 0.68 |
| VPS26B | 11q25 | 0.37 | 0.46 |
| RUBCNL | 13q14.13 | 0.33 | 0.39 |
| CEP290 | 12q21.32 | 0.37 | 0.32 |
| CHD9 | 16q12.2 | 0.36 | 0.35 |
| SPG11 | 15q21.1 | 0.41 | 0.47 |
| CFAP43 | 10q25.1 | 0.35 | 0.46 |
| ALPK1 | 4q25 | 0.33 | 0.34 |
| PLCD3 | 17q21.31 | -0.31 | -0.47 |
| MTERF2 | 12q23.3 | 0.33 | 0.35 |
| CPEB4 | 5q35.2 | 0.32 | 0.39 |
| CXXC4 | 4q24 | 0.44 | 0.36 |
| WDR82 | 3p21.2 | 0.34 | 0.35 |
| MAST4 | 5q12.3 | 0.35 | 0.41 |
| TMEM150C | 4q21.22 | 0.31 | 0.33 |
| EEF1A1P9 | 4q24 | 0.38 | 0.31 |
| TUBA3D | 2q21.1 | 0.36 | 0.57 |
| KDM7A | 7q34 | 0.53 | 0.49 |
| C16ORF71 | 16p13.3 | 0.31 | 0.41 |
| KIF18B | 17q21.31 | -0.33 | -0.35 |
| EME1 | 17q21.33 | -0.34 | -0.35 |
| TMEM199 | 17q11.2 | -0.34 | -0.4 |
| TDRD3 | 13q21.2 | 0.32 | 0.4 |
| PLA2G12A | 4q25 | 0.36 | 0.34 |
| CDADC1 | 13q14.2 | 0.34 | 0.43 |
| NUAK2 | 1q32.1 | -0.31 | -0.38 |
| TIRAP | 11q24.2 | 0.36 | 0.41 |
| SBF2 | 11p15.4 | 0.34 | 0.33 |
| TMEM123 | 11q22.2 | 0.31 | 0.4 |
| WDR31 | 9q32 | 0.43 | 0.41 |
| CNOT6L | 4q21.1 | 0.45 | 0.51 |
| GAS2L2 | 17q12 | 0.31 | 0.46 |
| RNASEH1 | 2p25.3 | -0.33 | -0.37 |
| FCHO2 | 5q13.2 | 0.31 | 0.34 |
| CREB3L4 | 1q21.3 | 0.42 | 0.44 |
| LYSMD3 | 5q14.3 | 0.35 | 0.4 |
| FAM210B | 20q13.2 | 0.33 | 0.41 |
| ARHGEF3 | 3p14.3 | 0.49 | 0.55 |
| ZMYND19 | 9q34.3 | -0.31 | -0.36 |
| DNAL1 | 14q24.3 | 0.38 | 0.41 |
| COG3 | 13q14.13 | 0.42 | 0.49 |
| TAS2R1 | 5p15.31 | 0.34 | 0.39 |
| CCM2 | 7p13 | -0.33 | -0.4 |
| RNF141 | 11p15.4\|11p15 | 0.34 | 0.43 |
| DNMBP-AS1 | 10q24.2 | 0.43 | 0.46 |
| CRISPLD1 | 8q21.13 | 0.39 | 0.39 |
| RSPH4A | 6q22.1 | 0.35 | 0.49 |
| ISOC1 | 5q23.3 | 0.55 | 0.64 |
| ECT2L | 6q24.1 | 0.32 | 0.47 |
| TVP23B | 17p11.2 | 0.4 | 0.35 |
| CRYL1 | 13q12.11 | 0.45 | 0.49 |
| ROPN1L | 5p15.2 | 0.32 | 0.45 |
| FSD1L | 9q31.2 | 0.32 | 0.39 |
| SEPSECS | 4p15.2 | 0.36 | 0.43 |
| ABHD5 | 3p21.33 | 0.37 | 0.38 |
| CEP83 | 12q22 | 0.41 | 0.43 |
| IRAK4 | 12q12 | 0.31 | 0.37 |
| CFAP57 | 1p34.2 | 0.35 | 0.38 |
| AADAT | 4q33 | 0.4 | 0.52 |
| TUBE1 | 6q21 | 0.41 | 0.36 |
| LEF1 | 4q25 | 0.5 | 0.58 |
| LRRC71 | 1q23.1 | 0.31 | 0.48 |
| PLCE1 | 10q23.33 | 0.48 | 0.6 |
| SRARP | 1p36.13 | 0.37 | 0.69 |
| OBSCN | 1q42.13 | -0.31 | -0.46 |
| GOLM1 | 9q21.33 | 0.33 | 0.42 |
| MYCBPAP | 17q21.33 | 0.44 | 0.49 |
| FAM198B | 4q32.1 | 0.39 | 0.56 |
| ARL6 | 3q11.2 | 0.34 | 0.34 |
| KIAA1109 | 4q27 | 0.41 | 0.32 |
| PRKAG2 | 7q36.1 | 0.44 | 0.35 |
| TMEM117 | 12q12 | 0.4 | 0.46 |
| REV1 | 2q11.2 | 0.42 | 0.42 |
| SFMBT1 | 3p21.1 | -0.31 | -0.33 |
| UBE2J1 | 6q15 | 0.42 | 0.52 |
| HSDL2 | 9q32 | 0.49 | 0.47 |
| NOA1 | 4q12 | 0.37 | 0.31 |
| NTPCR | 1q42.2 | 0.34 | 0.35 |
| PHF7 | 3p21.1 | 0.34 | 0.37 |
| ARMCX3 | Xq22.1 | 0.35 | 0.34 |
| UFM1 | 13q13.3 | 0.42 | 0.51 |
| GFM2 | 5q13.3 | 0.43 | 0.38 |
| AZIN1 | 8q22.3 | 0.31 | 0.36 |
| GET4 | 7p22.3 | -0.31 | -0.38 |
| MIA2 | 14q13.2 | 0.4 | 0.33 |
| TMBIM4 | 12q14.3 | 0.44 | 0.47 |
| ASB1 | 2q37.3 | -0.36 | -0.5 |
| SARAF | 8p12 | 0.32 | 0.32 |
| PHYHIPL | 10q21.1 | 0.4 | 0.4 |
| ACSL5 | 10q25.2 | 0.37 | 0.53 |
| FAM120B | 6q27 | 0.32 | 0.31 |
| LCA5L | 21q22.2 | 0.39 | 0.49 |
| GALNT7 | 4q34.1 | 0.33 | 0.41 |
| ZNF295-AS1 | 21q22.3 | 0.31 | 0.38 |
| UMODL1-AS1 | 21q22.3 | 0.35 | 0.47 |
| TUBB6 | 18p11.21 | -0.36 | -0.52 |
| ZGPAT | 20q13.33 | -0.31 | -0.41 |
| GLIS2 | 16p13.3 | -0.33 | -0.35 |
| CAPS2 | 12q21.1-q21.2 | 0.32 | 0.34 |
| LNX1 | 4q12 | 0.48 | 0.57 |
| TUBA1C | 12q13.12 | -0.31 | -0.35 |
| CGNL1 | 15q21.3 | 0.39 | 0.45 |
| CFAP300 | 11q22.1 | 0.38 | 0.39 |
| CCDC160 | Xq26.2 | 0.49 | 0.62 |
| CIPC | 14q24.3 | 0.41 | 0.46 |
| SERINC2 | 1p35.2 | 0.41 | 0.55 |
| ZNF385B | 2q31.2-q31.3 | 0.45 | 0.34 |
| LINC02418 | 12q24.33 | 0.31 | 0.57 |
| TMEM232 | 5q22.1 | 0.33 | 0.45 |
| CPXCR1 | Xq21.31 | 0.33 | 0.38 |
| CCDC144CP | 17p11.2 | 0.33 | 0.34 |
| CHIC1 | Xq13.2 | 0.33 | 0.48 |
| STX18 | 4p16.3-p16.2 | 0.52 | 0.61 |
| POC1B | 12q21.33 | 0.72 | 0.68 |
| BORCS7 | 10q24.32 | 0.58 | 0.58 |
| ROPN1B | 3q21.2 | 0.31 | 0.37 |
| ZCWPW2 | 3p24.1 | 0.43 | 0.4 |
| NEK10 | 3p24.1 | 0.41 | 0.41 |
| IL20RA | 6q23.3 | 0.42 | 0.58 |
| TRIM34 | 11p15.4 | 0.32 | 0.35 |
| CSNK1G1 | 15q22.31 | 0.32 | 0.34 |
| B4GALNT3 | 12p13.33 | 0.46 | 0.49 |
| GSAP | 7q11.23 | 0.4 | 0.49 |
| SUGT1P3 | 13q14.11 | 0.45 | 0.55 |
| FAM181A-AS1 | 14q32.12 | 0.34 | 0.39 |
| SAXO2 | 15q25.2 | 0.39 | 0.48 |
| DNAJC10 | 2q32.1 | 0.42 | 0.53 |
| CCSER2 | 10q23.1 | 0.42 | 0.46 |
| PLEKHA5 | 12p12.3 | 0.51 | 0.58 |
| THAP6 | 4q21.1 | 0.38 | 0.35 |
| USP53 | 4q26 | 0.56 | 0.49 |
| SPATA6 | 1p33 | 0.39 | 0.35 |
| LZTFL1 | 3p21.31 | 0.39 | 0.43 |
| ARL15 | 5q11.2 | 0.34 | 0.39 |
| MANSC1 | 12p13.2 | 0.33 | 0.53 |
| BTN2A3P | 6p22.2 | 0.44 | 0.35 |
| EPDR1 | 7p14.1 | 0.57 | 0.41 |
| BTG4 | 11q23.1 | 0.37 | 0.31 |
| TET2 | 4q24 | 0.41 | 0.42 |
| AHI1 | 6q23.3 | 0.46 | 0.44 |
| KLHL28 | 14q21.2 | 0.43 | 0.31 |
| ZNF280D | 15q21.3 | 0.51 | 0.49 |
| TRPM7 | 15q21.2 | 0.34 | 0.38 |
| NUP62CL | Xq22.3 | 0.35 | 0.43 |
| VPS13C | 15q22.2 | 0.31 | 0.34 |
| WBP1L | 10q24.32 | 0.31 | 0.34 |
| LRRC49 | 15q23 | 0.43 | 0.46 |
| PIH1D2 | 11q23.1 | 0.32 | 0.49 |
| FAM46C | 1p12 | 0.36 | 0.49 |
| SNRK | 3p22.1 | 0.37 | 0.38 |
| TMEM104 | 17q25.1 | -0.33 | -0.35 |
| PALMD | 1p21.2 | 0.56 | 0.56 |
| LINC00908 | 18q23 | 0.31 | 0.42 |
| TMEM260 | 14q22.3 | 0.45 | 0.46 |
| CEP120 | 5q23.2 | 0.42 | 0.47 |
| LPCAT2 | 16q12.2 | 0.42 | 0.56 |
| TTC12 | 11q23.2 | 0.32 | 0.52 |
| GID8 | 20q13.33 | -0.33 | -0.4 |
| 2-Mar | 1q41 | 0.4 | 0.49 |
| STX17 | 9q31.1 | 0.31 | 0.36 |
| OXR1 | 8q23.1 | 0.62 | 0.55 |
| ANO1 | 11q13.3 | 0.66 | 0.72 |
| ARHGAP17 | 16p12.1 | 0.37 | 0.47 |
| ARMC4 | 10p12.1 | 0.33 | 0.32 |
| ELP3 | 8p21.1 | 0.56 | 0.56 |
| NBPF15 | 1q21.1 | 0.58 | 0.33 |
| SLC25A36 | 3q23 | 0.44 | 0.39 |
| BBS7 | 4q27 | 0.31 | 0.31 |
| RCBTB1 | 13q14.2 | 0.39 | 0.36 |
| RAVER2 | 1p31.3 | 0.34 | 0.39 |
| SLC47A1 | 17p11.2 | 0.62 | 0.66 |
| MRGBP | 20q13.33 | -0.41 | -0.59 |
| ZNF654 | 3p11.1 | 0.35 | 0.34 |
| TBC1D19 | 4p15.2 | 0.35 | 0.33 |
| PI4K2B | 4p15.2 | 0.41 | 0.46 |
| TMEM144 | 4q32.1 | 0.38 | 0.43 |
| SLC39A9 | 14q24.1 | 0.36 | 0.38 |
| NIPSNAP3B | 9q31.1 | 0.38 | 0.31 |
| STRBP | 9q33.3 | 0.35 | 0.45 |
| HJURP | 2q37.1 | -0.31 | -0.35 |
| TTC23L | 5p13.2 | 0.32 | 0.45 |
| SLC35E3 | 12q15 | 0.38 | 0.4 |
| SMPD3 | 16q22.1 | 0.31 | 0.5 |
| DNAH3 | 16p12.3 | 0.33 | 0.4 |
| GALNT10 | 5q33.2 | 0.34 | 0.54 |
| CEP162 | 6q14.2-q14.3 | 0.33 | 0.46 |
| MTMR8 | Xq11.2 | 0.38 | 0.39 |
| CPEB3 | 10q23.32 | 0.36 | 0.48 |
| THUMPD1 | 16p12.3 | 0.31 | 0.45 |
| ICK | 6p12.1 | 0.36 | 0.37 |
| ENPP4 | 6p21.1 | 0.38 | 0.45 |
| ALKAL2 | 2p25.3 | 0.33 | 0.5 |
| SLC48A1 | 12q13.11 | -0.31 | -0.37 |
| DDX27 | 20q13.13 | -0.4 | -0.51 |
| RUFY2 | 10q21.3 | 0.37 | 0.35 |
| MAP7D1 | 1p34.3 | -0.35 | -0.37 |
| SHANK2 | 11q13.3-q13.4 | 0.42 | 0.51 |
| CNKSR3 | 6q25.2 | 0.31 | 0.31 |
| TPX2 | 20q11.21 | -0.35 | -0.43 |
| INAVA | 1q32.1 | -0.34 | -0.4 |
| WDFY3 | 4q21.23 | 0.39 | 0.36 |
| PLCH1 | 3q25.31 | 0.41 | 0.58 |
| CFAP44 | 3q13.2 | 0.38 | 0.39 |
| TMEM26 | 10q21.2 | 0.55 | 0.49 |
| CABCOCO1 | 10q21.2 | 0.31 | 0.53 |
| ZNF415 | 19q13.42 | 0.48 | 0.51 |
| LMBRD1 | 6q13 | 0.38 | 0.31 |
| PALLD | 4q32.3 | 0.52 | 0.55 |
| KDM4B | 19p13.3 | 0.45 | 0.49 |
| DOPEY1 | 6q14.1 | 0.31 | 0.38 |
| LRP2BP | 4q35.1 | 0.37 | 0.42 |
| MON2 | 12q14.1 | 0.42 | 0.35 |
| ZNF609 | 15q22.31 | 0.34 | 0.37 |
| ENKUR | 10p12.1 | 0.33 | 0.41 |
| EOGT | 3p14.1 | 0.31 | 0.41 |
| MBOAT1 | 6p22.3 | 0.46 | 0.46 |
| ARMC3 | 10p12.2 | 0.36 | 0.48 |
| UHRF1BP1L | 12q23.1 | 0.36 | 0.33 |
| VWA8 | 13q14.11 | 0.62 | 0.59 |
| EXPH5 | 11q22.3 | 0.34 | 0.69 |
| ARHGAP26 | 5q31.3 | 0.5 | 0.62 |
| TTLL5 | 14q24.3 | 0.31 | 0.33 |
| SPART | 13q13.3 | 0.31 | 0.33 |
| CLASP2 | 3p22.3 | 0.41 | 0.39 |
| GPRC5C | 17q25.1 | -0.31 | -0.33 |
| COLGALT2 | 1q25.3 | 0.38 | 0.39 |
| LRRC70 | 5q12.1 | 0.32 | 0.31 |
| STOX1 | 10q22.1 | 0.43 | 0.36 |
| RNF114 | 20q13.13 | -0.32 | -0.47 |
| DCUN1D4 | 4q12 | 0.33 | 0.33 |
| ERBIN | 5q12.3 | 0.37 | 0.4 |
| FCHO1 | 19p13.11 | -0.33 | -0.54 |
| FRMD4B | 3p14.1 | 0.36 | 0.43 |
| MYO5C | 15q21.2 | 0.4 | 0.49 |
| METAP1 | 4q23 | 0.48 | 0.41 |
| RGL1 | 1q25.3 | 0.34 | 0.34 |
| FBXL7 | 5p15.1 | 0.33 | 0.44 |
| TCAIM | 3p21.31 | 0.36 | 0.34 |
| FAM120A | 9q22.31 | 0.31 | 0.39 |
| RAB5IF | 20q11.23 | -0.38 | -0.43 |
| BCAP29 | 7q22.3 | 0.33 | 0.31 |
| TBC1D12 | 10q23.33 | 0.45 | 0.34 |
| PLCB1 | 20p12.3 | 0.61 | 0.63 |
| COBL | 7p12.1 | 0.33 | 0.58 |
| CFAP206 | 6q15 | 0.34 | 0.4 |
| TMEM218 | 11q24.2 | 0.35 | 0.38 |
| DDHD2 | 8p11.23 | 0.31 | 0.43 |
| PPIP5K2 | 5q21.1 | 0.34 | 0.33 |
| MCF2L | 13q34 | 0.36 | 0.47 |
| DNMBP | 10q24.2 | 0.31 | 0.44 |
| RPGRIP1L | 16q12.2 | 0.36 | 0.33 |
| ARHGEF12 | 11q23.3 | 0.34 | 0.39 |
| UFL1 | 6q16.1 | 0.37 | 0.42 |
| FRYL | 4p11 | 0.41 | 0.49 |
| SMG5 | 1q22 | -0.33 | -0.42 |
| SIK3 | 11q23.3 | 0.45 | 0.41 |
| NCS1 | 9q34.11 | -0.34 | -0.44 |
| GRIP1 | 12q14.3 | 0.4 | 0.4 |
| FAM214A | 15q21.2-q21.3 | 0.57 | 0.65 |
| SLC35A3 | 1p21.2 | 0.36 | 0.38 |
| KIAA0825 | 5q15 | 0.47 | 0.48 |
| ABCA5 | 17q24.3 | 0.32 | 0.48 |
| CES3 | 16q22.1 | 0.31 | 0.47 |
| GPCPD1 | 20p12.3 | 0.33 | 0.41 |
| HEY2 | 6q22.31 | 0.5 | 0.38 |
| TMEM131 | 2q11.2 | 0.32 | 0.45 |
| KCTD2 | 17q25.1 | -0.32 | -0.37 |
| CDK20 | 9q22.1 | 0.38 | 0.47 |
| DDAH1 | 1p22.3 | 0.31 | 0.48 |
| RUNDC3B | 7q21.12 | 0.43 | 0.46 |
| PPIL6 | 6q21 | 0.31 | 0.36 |
| SH3BP1 | 22q13.1 | -0.35 | -0.52 |
| LRRC6 | 8q24.22 | 0.31 | 0.44 |
| STX12 | 1p35.3 | 0.35 | 0.34 |
| SH3BP4 | 2q37.2 | 0.32 | 0.4 |
| SGK3 | 8q13.1 | 0.45 | 0.45 |
| LOC154761 | 7q35 | 0.36 | 0.31 |
| SLAIN1 | 13q22.3 | 0.44 | 0.41 |
| IQUB | 7q31.32 | 0.37 | 0.42 |
| TCAF2 | 7q35 | 0.38 | 0.35 |
| CTAGE10P | 13q14.2 | 0.45 | 0.33 |
| SLC26A4-AS1 | 7q22.3 | 0.31 | 0.33 |
| TRIQK | 8q22.1 | 0.37 | 0.32 |
| ZBTB38 | 3q23 | 0.33 | 0.35 |
| TM9SF3 | 10q24.1 | 0.37 | 0.36 |
| MDM1 | 12q15 | 0.43 | 0.43 |
| TMEM167B | 1p13.3 | 0.46 | 0.56 |
| LAMP5 | 20p12.2 | 0.42 | 0.36 |
| PRPF6 | 20q13.33 | -0.31 | -0.37 |
| SEMA3G | 3p21.1 | 0.4 | 0.36 |
| AK7 | 14q32.2 | 0.33 | 0.35 |
| OTUD7B | 1q21.2 | 0.47 | 0.39 |
| PRDM10 | 11q24.3 | 0.33 | 0.33 |
| DTWD1 | 15q21.2 | 0.44 | 0.49 |
| C14ORF28 | 14q21.2 | 0.32 | 0.36 |
| FAM47E | 4q21.1 | 0.41 | 0.51 |
| ENTPD7 | 10q24.2 | 0.36 | 0.31 |
| FAM13C | 10q21.1 | 0.57 | 0.59 |
| GOLGA6L10 | 15q25.2 | 0.33 | 0.33 |
| SNX14 | 6q14.3 | 0.44 | 0.32 |
| RALGAPA1 | 14q13.2 | 0.47 | 0.37 |
| ABHD6 | 3p14.3 | 0.31 | 0.33 |
| AK9 | 6q21 | 0.43 | 0.53 |
| NDRG2 | 14q11.2 | 0.5 | 0.5 |
| GALNT16 | 14q24.1 | 0.31 | 0.34 |
| KIAA1143 | 3p21.31 | 0.31 | 0.31 |
| CTAGE11P | 13q22.2 | 0.34 | 0.36 |
| PPM1H | 12q14.1-q14.2 | 0.36 | 0.5 |
| ERMN | 2q24.1 | 0.53 | 0.64 |
| PLEKHH1 | 14q24.1 | 0.56 | 0.49 |
| GMNC | 3q28 | 0.32 | 0.56 |
| LOC90246 | 3q21.3 | 0.41 | 0.47 |
| TTC8 | 14q31.3 | 0.51 | 0.46 |
| ODF2L | 1p22.3 | 0.38 | 0.42 |
| MTUS1 | 8p22 | 0.43 | 0.49 |
| COG6 | 13q14.11 | 0.37 | 0.39 |
| KIAA1324 | 1p13.3 | 0.75 | 0.72 |
| CC2D2A | 4p15.32 | 0.36 | 0.38 |
| CEP126 | 11q22.1 | 0.33 | 0.48 |
| SPATS1 | 6p21.1 | 0.41 | 0.46 |
| CCDC191 | 3q13.31 | 0.34 | 0.4 |
| TMEM181 | 6q25.3 | 0.41 | 0.32 |
| TRMT9B | 8p22 | 0.47 | 0.43 |
| SLAIN2 | 4p11 | 0.4 | 0.55 |
| DPP10 | 2q14.1 | 0.34 | 0.36 |
| TMEM41A | 3q27.2 | -0.32 | -0.4 |
| CCDC146 | 7q11.23 | 0.45 | 0.53 |
| DHX37 | 12q24.31 | -0.34 | -0.38 |
| LEMD2 | 6p21.31 | -0.32 | -0.31 |
| TRIB3 | 20p13 | -0.32 | -0.51 |
| ANKRA2 | 5q13.2 | 0.41 | 0.4 |
| GATAD1 | 7q21.2 | 0.34 | 0.36 |
| RBM24 | 6p22.3 | 0.43 | 0.55 |
| SLC46A2 | 9q32 | 0.32 | 0.45 |
| LPCAT4 | 15q14 | -0.31 | -0.34 |
| JAZF1 | 7p15.2-p15.1 | 0.45 | 0.46 |
| IL33 | 9p24.1 | 0.35 | 0.49 |
| DAGLB | 7p22.1 | -0.32 | -0.39 |
| C16ORF46 | 16q23.2 | 0.34 | 0.42 |
| PRR15 | 7p14.3 | 0.42 | 0.45 |
| ATXN7L1 | 7q22.3 | 0.31 | 0.48 |
| CFAP65 | 2q35 | 0.31 | 0.49 |
| OSTC | 4q25 | 0.35 | 0.33 |
| CFAP299 | 4q21.21 | 0.31 | 0.48 |
| KMT2C | 7q36.1 | 0.46 | 0.4 |
| SLC39A6 | 18q12.2 | 0.41 | 0.41 |
| SPDEF | 6p21.31 | 0.46 | 0.68 |
| EEF1AKMT3 | 12q14.1 | 0.43 | 0.37 |
| MTHFD1L | 6q25.1 | -0.32 | -0.41 |
| NIPSNAP3A | 9q31.1 | 0.45 | 0.45 |
| HEATR5A | 14q12 | 0.35 | 0.32 |
| SAMHD1 | 20q11.23 | 0.35 | 0.38 |
| PNKD | 2q35 | -0.33 | -0.36 |
| TIPARP | 3q25.31 | 0.33 | 0.31 |
| REXO2 | 11q23.2 | 0.31 | 0.34 |
| LRIG1 | 3p14.1 | 0.33 | 0.55 |
| POLDIP2 | 17q11.2 | -0.31 | -0.32 |
| CFAP61 | 20p11.23 | 0.31 | 0.39 |
| WIPI2 | 7p22.1 | -0.31 | -0.35 |
| GMEB2 | 20q13.33 | -0.36 | -0.42 |
| FBXL5 | 4p15.32 | 0.38 | 0.45 |
| MYLK3 | 16q11.2 | 0.38 | 0.54 |
| ARFGAP3 | 22q13.2 | 0.5 | 0.47 |
| EHF | 11p13 | 0.35 | 0.42 |
| LINC00964 | 8q24.13 | 0.37 | 0.41 |
| MARVELD3 | 16q22.2 | 0.35 | 0.39 |
| CCDC129 | 7p14.3 | 0.37 | 0.55 |
| SEMA3D | 7q21.11 | 0.42 | 0.51 |
| PAN3 | 13q12.2 | 0.31 | 0.38 |
| CEP85L | 6q22.31 | 0.44 | 0.31 |
| SH3YL1 | 2p25.3 | 0.58 | 0.54 |
| KRTAP5-1 | 11p15.5 | 0.35 | 0.37 |
| GK5 | 3q23 | 0.39 | 0.37 |
| SPECC1 | 17p11.2 | 0.33 | 0.37 |
| SERPINA11 | 14q32.13 | 0.35 | 0.52 |
| ACAD8 | 11q25 | 0.35 | 0.38 |
| TIFA | 4q25 | 0.37 | 0.32 |
| TMEM189 | 20q13.13 | -0.32 | -0.38 |
| FILIP1 | 6q14.1 | 0.35 | 0.46 |
| INTU | 4q28.1 | 0.35 | 0.42 |
| NAAA | 4q21.1 | 0.39 | 0.44 |
| C5AR2 | 19q13.32 | 0.43 | 0.56 |
| ARFIP1 | 4q31.3 | 0.35 | 0.49 |
| LINC00475 | 9q22.31 | 0.33 | 0.39 |
| SESN1 | 6q21 | 0.47 | 0.48 |
| PDCD4 | 10q25.2 | 0.5 | 0.57 |
| BCLAF3 | Xp22.12 | 0.41 | 0.45 |
| DIMT1 | 5q12.1 | 0.37 | 0.37 |
| GOLIM4 | 3q26.2 | 0.35 | 0.34 |
| ADAMTSL1 | 9p22.2-p22.1 | 0.35 | 0.4 |
| ZNF816 | 19q13.41 | 0.37 | 0.48 |
| ELOVL5 | 6p12.1 | 0.52 | 0.5 |
| AKAP14 | Xq24 | 0.31 | 0.44 |
| ZNF491 | 19p13.2 | 0.32 | 0.37 |
| HAUS8 | 19p13.11 | -0.37 | -0.38 |
| SPCS3 | 4q34.2 | 0.36 | 0.33 |
| SHISA6 | 17p12 | 0.31 | 0.54 |
| ZFAND4 | 10q11.22 | 0.34 | 0.31 |
| TRMT10A | 4q23 | 0.46 | 0.35 |
| USP54 | 10q22.2 | 0.31 | 0.38 |
| SLCO3A1 | 15q26.1 | -0.34 | -0.43 |
| FMO6P | 1q24.3 | 0.37 | 0.57 |
| WDR63 | 1p22.3 | 0.31 | 0.39 |
| SYTL4 | Xq22.1 | 0.52 | 0.49 |
| ERICH3 | 1p31.1 | 0.42 | 0.63 |
| ZCCHC4 | 4p15.2 | 0.33 | 0.39 |
| CCDC113 | 16q21 | 0.35 | 0.48 |
| MYLIP | 6p22.3 | 0.45 | 0.38 |
| LMNTD1 | 12p12.1 | 0.33 | 0.4 |
| UBXN10 | 1p36.12 | 0.37 | 0.57 |
| ZNF704 | 8q21.13 | 0.45 | 0.45 |
| DGKH | 13q14.11 | 0.42 | 0.32 |
| CCDC122 | 13q14.11 | 0.35 | 0.34 |
| SNX8 | 7p22.3 | -0.32 | -0.45 |
| ANAPC4 | 4p15.2 | 0.38 | 0.53 |
| NRBP1 | 2p23.3 | -0.31 | -0.38 |
| PSAT1 | 9q21.2 | -0.31 | -0.4 |
| SLC40A1 | 2q32.2 | 0.46 | 0.63 |
| EML5 | 14q31.3 | 0.47 | 0.36 |
| CFAP161 | 15q25.1 | 0.36 | 0.47 |
| DNAAF4 | 15q21.3 | 0.39 | 0.42 |
| PLA2G2C | 1p36.12 | 0.36 | 0.41 |
| FAM86B2 | 8p23.1 | 0.4 | 0.35 |
| LIMS3 | 2q13 | 0.33 | 0.39 |
| MCUR1 | 6p23 | -0.31 | -0.42 |
| NEUROG2 | 4q25 | 0.42 | 0.37 |
| NPAS3 | 14q13.1 | 0.61 | 0.63 |
| MCCC2 | 5q13.2 | 0.35 | 0.47 |
| MBOAT2 | 2p25.1 | -0.32 | -0.37 |
| TOR3A | 1q25.2 | 0.35 | 0.31 |
| RNF25 | 2q35 | -0.31 | -0.33 |
| SOX17 | 8q11.23 | 0.46 | 0.59 |
| AZI2 | 3p24.1 | 0.38 | 0.39 |
| IKZF5 | 10q26.13 | 0.41 | 0.37 |
| GREM2 | 1q43 | 0.46 | 0.49 |
| TMEM168 | 7q31.1 | 0.39 | 0.39 |
| INF2 | 14q32.33 | -0.31 | -0.38 |
| SUDS3 | 12q24.23 | 0.32 | 0.38 |
| ZDHHC6 | 10q25.2 | 0.34 | 0.4 |
| PCNX4 | 14q23.1 | 0.43 | 0.35 |
| CTAGE1 | 18q11.2 | 0.46 | 0.36 |
| CREB3L2 | 7q33 | 0.44 | 0.41 |
| C6ORF106 | 6p21.31 | -0.31 | -0.35 |
| EFCAB6 | 22q13.2-q13.31 | 0.33 | 0.37 |
| PORCN | Xp11.23 | 0.32 | 0.44 |
| YTHDC2 | 5q22.2 | 0.33 | 0.45 |
| USP46 | 4q12 | 0.35 | 0.32 |
| SLC30A5 | 5q13.1-q13.2 | 0.31 | 0.36 |
| TMEM135 | 11q14.2 | 0.35 | 0.33 |
| TMEM178A | 2p22.1 | 0.31 | 0.37 |
| RILPL2 | 12q24.31 | 0.31 | 0.44 |
| DNAH10 | 12q24.31 | 0.33 | 0.42 |
| FBXO36 | 2q36.3 | 0.4 | 0.46 |
| CCDC148 | 2q24.1 | 0.4 | 0.48 |
