# Supplementary material for: Comprehensive bioinformatics analysis of acquired progesterone resistance in endometrial cancer cell line
Source: J Transl Med. 2019 Feb 27;17:58. doi: 10.1186/s12967-019-1814-6 (PMC6391799; doi:10.1186/s12967-019-1814-6)
Supplement: Supplementary file 6 — Additional file 6: Table S6. Distinguishing and validation of 36 genes that were overlapping of PGR co-expression genes and DEGs. [file 12967_2019_1814_MOESM6_ESM.docx]

**Additional Table S6**. Distinguishing and validation of 36 genes that were overlapping of PGR co-expression genes and DEGs. The second column is PGR co-expression analysis of 549 TCGA endometrial carcinoma by using cBioPortal website ([www.cbioportal.org](http://www.cbioportal.org)). The third column shows the result of microarray analysis between parental Ishikawa and IshikawaPR cell. The fourth column is the validation by using GSE17025 dataset. (FC: Fold change).

|  | **PGR co-expression (TCGA)** | | **microarray analysis** | | **GSE17025** | | | |
| --- | --- | --- | --- | --- | --- | --- | --- | --- |
|  |  |  |  |  | **Pearson Score** | | **Spearman Score** | |
| gene symbol | Pearson Score | Spearman Score | LogFC | Log(p-value) | r | p-value | r | p-value |
| ANO1 | 0.66 | 0.72 | -10.78 | 8.69 | 0.611 | <0.01 | 0.719 | <0.01 |
| PLCB1 | 0.61 | 0.63 | -4.25 | 5.87 | 0.354 | <0.01 | 0.375 | <0.01 |
| NPAS3 | 0.61 | 0.63 | -9.40 | 8.08 | 0.606 | <0.01 | 0.648 | <0.01 |
| SH3YL1 | 0.58 | 0.54 | -4.40 | 6.41 | 0.416 | <0.01 | 0.447 | <0.01 |
| ZNF415 | 0.48 | 0.51 | -5.05 | 5.56 | 0.186 | ns | 0.232 | <0.1 |
| SOX17 | 0.46 | 0.59 | -4.03 | 5.83 | 0.444 | <0.01 | 0.598 | <0.01 |
| SLC40A1 | 0.46 | 0.63 | -5.65 | 7.73 | 0.417 | <0.01 | 0.489 | <0.01 |
| CCDC146 | 0.45 | 0.53 | -4.45 | 8.30 | 0.255 | <0.01 | 0.429 | <0.01 |
| SLAIN1 | 0.44 | 0.41 | -6.77 | 3.35 | 0.187 | ns | 0.251 | <0.1 |
| RUNDC3B | 0.43 | 0.46 | -4.23 | 4.64 | 0.235 | <0.1 | 0.461 | <0.01 |
| CYP2J2 | 0.42 | 0.31 | -4.05 | 4.87 | 0.184 | ns | 0.263 | <0.01 |
| SEMA3D | 0.42 | 0.51 | -6.58 | 6.95 | 0.415 | <0.01 | 0.412 | <0.01 |
| FBXO36 | 0.4 | 0.46 | -4.77 | 4.77 | 0.094 | ns | 0.271 | <0.01 |
| CRISPLD1 | 0.39 | 0.39 | -4.09 | 4.42 | 0.434 | <0.01 | 0.441 | <0.01 |
| CGNL1 | 0.39 | 0.45 | -7.19 | 3.38 | 0.438 | <0.01 | 0.484 | <0.01 |
| ZNF204P | 0.39 | 0.4 | -7.21 | 2.77 | 0.104 | ns | 0.19 | ns |
| CDS1 | 0.38 | 0.4 | -4.32 | 4.18 | 0.207 | <0.1 | 0.308 | <0.01 |
| GNA14 | 0.38 | 0.47 | -5.49 | 9.59 | 0.150 | ns | 0.189 | ns |
| GRHL2 | 0.37 | 0.51 | -7.97 | 6.75 | -0.018 | ns | 0.043 | ns |
| PZP | 0.36 | 0.56 | -7.00 | 5.82 | 0.486 | <0.01 | 0.043 | ns |
| MARVELD3 | 0.35 | 0.39 | -4.64 | 1.85 | 0.113 | ns | -0.101 | ns |
| XIST | 0.34 | 0.33 | -8.91 | 8.47 | 0.282 | <0.01 | 0.307 | <0.01 |
| ARMC4 | 0.33 | 0.32 | -5.00 | 3.71 | -0.052 | ns | -0.017 | ns |
| DACH1 | 0.33 | 0.39 | -6.67 | 4.60 | 0.285 | <0.01 | 0.417 | <0.01 |
| CAPS2 | 0.32 | 0.34 | -4.43 | 8.55 | 0.145 | ns | 0.095 | ns |
| SLCO3A1 | -0.34 | -0.43 | 8.44 | 8.25 | -0.217 | <0.1 | -0.343 | <0.01 |
| ACAA2 | -0.34 | -0.43 | 4.27 | 7.24 | -0.175 | ns | -0.181 | ns |
| PAX8 | -0.31 | -0.35 | -5.58 | 6.45 | -0.314 | <0.01 | -0.364 | <0.01 |
| KIF1A | -0.31 | -0.36 | -8.90 | 7.25 | -0.114 | ns | -0.031 | ns |
| KIAA0825 | 0.47 | 0.48 | 4.06 | 3.64 | 0.030 | ns | 0.155 | ns |
| VIM | 0.45 | 0.4 | 11.81 | 6.18 | 0.586 | <0.01 | 0.542 | <0.01 |
| COL12A1 | 0.45 | 0.46 | 4.88 | 6.51 | 0.284 | <0.01 | 0.472 | <0.01 |
| RBM24 | 0.43 | 0.55 | 5.12 | 4.92 | 0.236 | <0.1 | 0.589 | <0.01 |
| ADAMTS8 | 0.42 | 0.42 | 6.92 | 5.81 | 0.180 | ns | 0.368 | <0.01 |
| AR | 0.41 | 0.52 | 4.89 | 6.61 | 0.319 | <0.01 | 0.459 | <0.01 |
| MYLK3 | 0.38 | 0.54 | 4.26 | 4.39 | 0.421 | <0.01 | 0.499 | <0.01 |
