# Supplementary material for: Comprehensive bioinformatics analysis of acquired progesterone resistance in endometrial cancer cell line
Source: J Transl Med. 2019 Feb 27;17:58. doi: 10.1186/s12967-019-1814-6 (PMC6391799; doi:10.1186/s12967-019-1814-6)

**Additional Figure S1**. The survival curves of 7 PGR co-expressed genes comparing the patients with high (red) and low (black) expression in endometrial cancer was plotted from Kaplan-Meier plotter database.


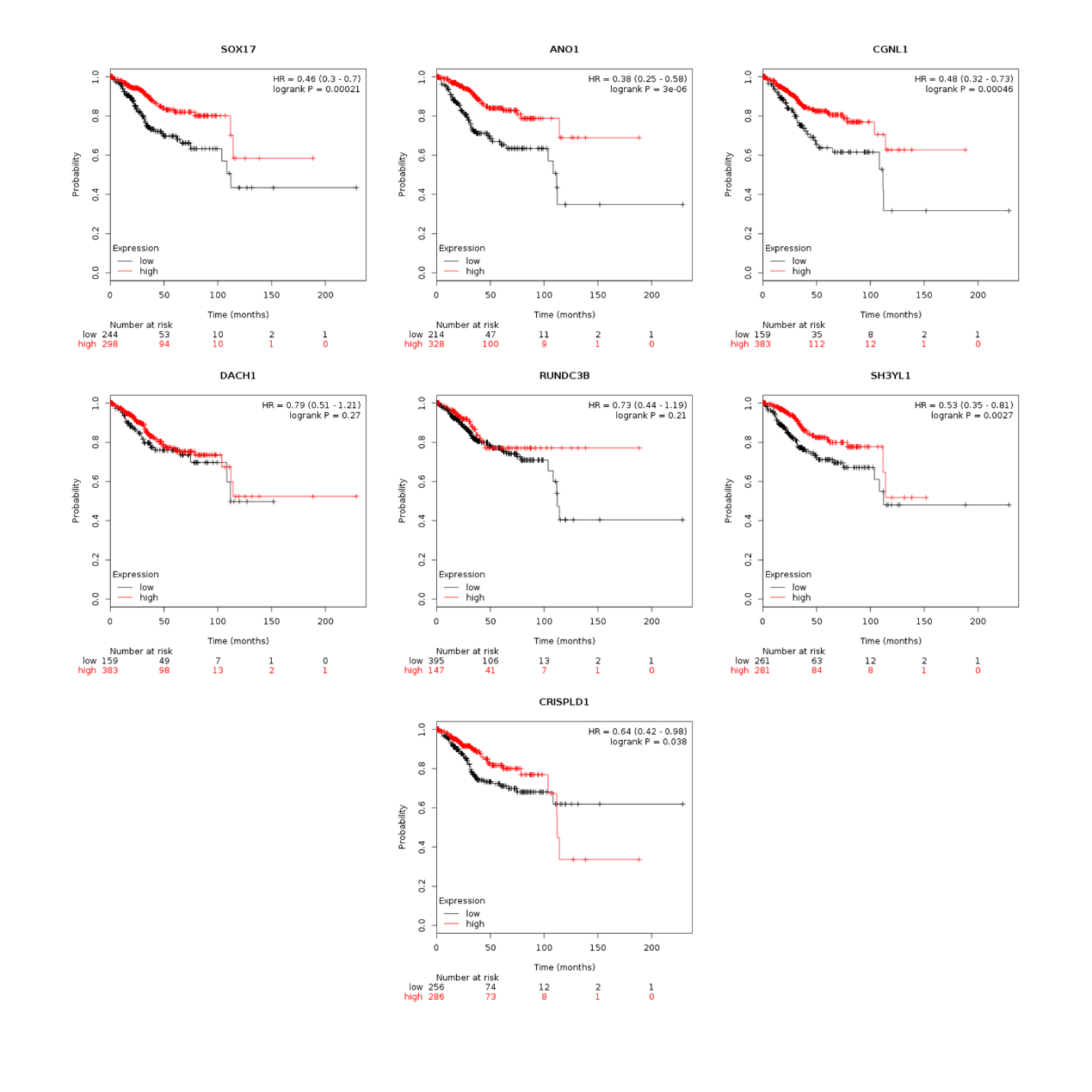

Supplement: Supplementary file 7 — Additional file 7: Figure S1. The survival curves of 7 PGR co-expressed genes comparing the patients with high (red) and low (black) expression in endometrial cancer. [file 12967_2019_1814_MOESM7_ESM.docx]
